# Today’s Adolescents Are More Satisfied With Being Single: Findings From a German Cohort-Sequential Study Among 14- to 40-Year-Olds

**DOI:** 10.1177/01461672241257139

**Published:** 2024-06-10

**Authors:** Tita Gonzalez Avilés, Janina Larissa Bühler, Naemi D. Brandt, Franz J. Neyer

**Affiliations:** 1 Friedrich Schiller University Jena, Germany; 2 Johannes Gutenberg University Mainz, Germany; 3 University of Hamburg, Germany

**Keywords:** singlehood, singlehood satisfaction, life satisfaction, cohort effects, historical change

## Abstract

In Western societies, singlehood has become increasingly normative over historical time. But whether singles are more satisfied nowadays remains unclear. In this preregistered cohort-sequential study, we analyzed data from 2,936 German participants (*M* = 21.01 years, *SD* = 7.60 years) from different birth cohorts. Singlehood satisfaction and life satisfaction were reported annually at two different time periods (2008-2011 and 2018-2021). This design allowed us to compare earlier-born and later-born singles during adolescence (14-20 years), emerging adulthood (24-30 years), and established adulthood (34-40 years). Results from multilevel growth-curve models indicated that adolescent singles born in 2001 to 2003 (vs. 1991-1993) were more often single and more satisfied with singlehood. No cohort-related differences emerged among emerging and established adults. Younger age and lower neuroticism predicted higher satisfaction, regardless of birth cohort. The results highlight the importance of considering both societal and individual factors to understand singles’ satisfaction.

Throughout the world, marriage rates have been declining over the past decades while divorce rates and single-person households are on the rise (EuroStat, 2024; OECD, 2022). While it is crucial to distinguish civil status, living arrangements, and relationship status (Mortelmans et al., 2023), these trends may collectively point toward a growing population of singles—defined herein as individuals not actively involved in a romantic relationship. At the same time, societal attitudes toward romantic relationships have evolved, fostering greater inclusivity and acceptance of diverse relationship types, such as living-apart-together relationships and prolonged singlehood (Lesthaeghe, 2014; Tillman et al., 2019). Consequently, being single has become more normative over historical time (Bühler & Nikitin, 2020). These sociohistorical changes may have contributed to a higher satisfaction with life and higher singlehood satisfaction among singles nowadays compared to previous generations.

We tested this assumption using data from a cohort-sequential study including singles from four birth cohorts (1971-1973, 1981-1983, 1991-1993, 2001-2003) who provided data on their satisfaction between the time periods 2008 to 2011 and 2018 to 2021. Furthermore, we tested moderating effects of gender, extraversion, and neuroticism on singles’ satisfaction.

## Satisfaction of Singles

In many Western industrialized societies, romantic relationships and marriage are highly valued, while singlehood is often viewed less favorably (DePaulo & Morris, 2005). This societal emphasis on partnership often results in discrimination and negative stereotypes against singles, who may be seen as failing a crucial developmental milestone. Indeed, single individuals not only face stereotypes of being less happy (DePaulo & Morris, 2005; Greitemeyer, 2009), but also on average report lower life satisfaction (Stahnke & Cooley, 2021) and less satisfaction with their relationship status (Poortman & Liefbroer, 2010) than those in a romantic relationship. In addition, people tend to become increasingly dissatisfied with their single life over time of being single (Oh et al., 2022). Singles also encounter significant stigma, with prejudice against them often perceived as more socially acceptable than against other marginalized groups, posing risks to their well-being (Fisher & Sakaluk, 2020).

However, the extent to which singles are satisfied likely depends on the broader context. Lifespan perspectives (Baltes et al., 2007; Bronfenbrenner, 1986; Schaie, 1965) have long postulated that human development is embedded within and influenced by broader societal, cultural, and historical contexts. For example, the contexts and norms regarding partnership and singlehood have undergone significant historical changes (Bühler & Nikitin, 2020), which may result in distinct satisfaction levels among singles from different birth cohorts.

## Singlehood Over Historical Time

Marriage rates have sharply declined since the 1970s across most Western industrialized countries, accompanied by a rise in divorce rates (OECD, 2022). Simultaneously, factors like increased life expectancy and prolonged education have alleviated societal pressure to settle into adult social roles (i.e., marriage and parenthood) right after entering adulthood (Arnett, 2000). Consequently, people nowadays are getting married later in life (OECD, 2022) and spending more time of their life being single (Eckhard, 2015).

Rising geographic mobility and increased access to social media have further contributed to greater exposure to and acceptance of diverse relationship types including unmarried cohabitation, divorce, and prolonged singlehood (Tillman et al., 2019). Moreover, cohorts born in later years have exhibited increased skepticism toward established social structures and to endorse individual freedom of choice and autonomy (Lesthaeghe, 2014) more strongly. Notably, when asked to describe important goals they pursued in their early 20s, earlier-born cohorts (born 1920-1925) tended to focus on more traditional developmental tasks such as getting married and starting a family, while later-born cohorts (born 1970-1975) emphasized more individualistic and self-oriented goals (Krings et al., 2008).

These sociohistorical changes could have contributed to a changing role of singlehood for satisfaction. Singles nowadays may feel less societal pressure to be in a romantic relationship and experience greater acceptance of being single. These changes, in turn, may lead to singles experiencing fewer negative stereotypes and discrimination and a stronger sense of autonomy, both of which associated to higher well-being (Byrne & Carr, 2005; DePaulo & Morris, 2005; Kislev, 2018). Indeed, there is some empirical support suggesting positive developments for singles’ well-being. Van Tilburg, Aartsen, and van der Pas (2015), for example, found that being divorced was associated with lower loneliness in 2012 among 54- to 65-year-olds compared to 1992. Similarly, the association between being single and loneliness became weaker among 40- to 85-year-olds between 2008 and 2014, and later-born singles were more satisfied with being single (Böger & Huxhold, 2020).

While these data show positive historical trends among middle-aged and older singles, they cannot speak to the levels of satisfaction of adolescents and younger adults. Notably, historical changes may be particularly relevant for younger singles, as romantic relationships gain prominence during adolescence (Gonzalez Avilés et al., 2021) and peak in the late 30s, a life stage often characterized by people becoming married and/or parents (Mehta et al., 2020). Findings from a recent study support this notion, indicating that midlife is an important changing point for the satisfaction of singles, showing that singles tend to be less satisfied before the age of 40, with an increase observed thereafter (Park et al., 2022). Limited research on historical changes among younger ages, such as a study by Scheling and Richter (2021), indicates a reduced emphasis on romantic partners for personal happiness among 17-year-olds in 2015 compared to 2000. However, the implications of these attitudinal shifts on the satisfaction of adolescent singles today and potential changes among adults up to age 40 remain unclear.

In addition to investigating how cohort differences relate to singles’ satisfaction *on average*, it is imperative to acknowledge the heterogeneity within singles (Girme et al., 2022). Specifically, satisfaction among singles can vary based on different individual factors including demographics and personality traits. In addition, the effects of these individual factors may fluctuate across different time periods, influenced by the ongoing interplay between individuals and their sociohistorical contexts (Baltes et al., 2007). To achieve a thorough understanding of singles’ satisfaction, it is important to examine both sociohistorical and individual factors along with their potential interactions.

## The Role of Age and Gender

Age-graded influences significantly shape human development by defining normative events and transitions associated with specific age phases (Baltes et al., 2007). These events and transition are often guided by societal expectations regarding developmental milestones for each life phase (Havighurst, 1972). Consequently, individuals may anticipate certain events simply because they are considered typical for their age.

In the context of romantic relationships, the prevalence and salience of romantic relationships increase over the first half of life. While adolescence (i.e., 10-18 years; Smetana et al., 2006) marks the period when most youth start dating, a substantial proportion remains single during this time (Gonzalez Avilés et al., 2021). As adolescents transition into emerging adulthood (i.e., 18-29 years; Nelson, 2021), however, the proportion of partnered individuals continuously increases (Seiffge-Krenke, 2003). Romantic relationships become even more prevalent and salient in established adulthood (i.e., 30-45 years; Mehta et al., 2020), a period that is characterized by first marriages and child births (OECD, 2022). Thus, being single becomes less normative across the first half of life, exposing individuals to potential negative stereotypes and discrimination for deviating from prevailing relationship norms. For example, 40-year-old singles are often perceived more negatively (e.g., more immature) than 25-year-old singles, as found in a U.S. sample (DePaulo & Morris, 2005). In addition, having never been married was associated with worse mental health among Korean individuals who were older (vs. younger) than 30 years (Lee et al., 2020).

Age-graded influences can systematically vary by gender (Baltes et al., 2007). Specifically, societal expectations, cultural norms, and social pressures related to age and relationship status might affect single men and single women differently. Marriage and parenthood are often regarded as central to adult women’s identities in many Western societies (Barnett & Hyde, 2001). Consequently, lack of a romantic relationship may pose a risk to the satisfaction of single women by defying prevailing gender norms, potentially resulting in increased stigmatization and discrimination. Qualitative studies show that single women often feel harshly judged and pressured to find a romantic partner and have children (Reynolds & Wetherell, 2003; Sharp & Ganong, 2011). However, other studies found no gender differences in personal discrimination (Byrne & Carr, 2005; Dupuis & Girme, 2023; Greitemeyer, 2009). The extent to which singlehood in adulthood poses a risk for the satisfaction of women compared to men remains an open question.

In the current study, we tested for age and gender differences in singles’ satisfaction. Grounded in lifespan perspectives, which propose that history- and age-related influences interact in shaping human development (Baltes et al., 2007), we expected age and gender differences to be weaker in more recent generations. This is because of the decreasing importance of meeting traditional milestones of adulthood (e.g., marriage and parenthood; Krings et al., 2008) and the increasing gender equality (Donnelly et al., 2016) observed over the last decades.

## The Role of Extraversion and Neuroticism

Differences in singles’ satisfaction could also be related to personality traits, particularly to extraversion and neuroticism. These two traits have been identified as the most robust predictors of subjective well-being among the Big Five personality traits (Heller et al., 2004) and are linked to diverse social experiences that could be particularly relevant to the context of singlehood, as described in the following.

*Extraversion*—the tendency to be socially active, outgoing, and energetic—is positively related to life satisfaction and marital satisfaction (Heller et al., 2004). In addition, more (vs. less) extraverted people report larger social networks and support (Ozer & Benet-Martínez, 2006), which may play an important role in fostering singles’ satisfaction. A recent study by Park, Impett, and MacDonald (2020) showed that singles who were more satisfied with their friendships reported greater life satisfaction and satisfaction with singlehood. *Neuroticism*—the tendency to feeling anxious, worried, and distressed—is related to lower life satisfaction (Heller et al., 2004) and lower relationship satisfaction (Weidmann et al., 2016). In addition, a study by Spielmann et al. (2013) showed that people with higher levels of neuroticism were more afraid of being single, which might translate into singles with higher neuroticism reporting lower satisfaction. Thus, we expected that these two traits matter for singles’ satisfaction, assuming a positive association with extraversion and a negative association with neuroticism.

These associations, however, may depend on the historical context, in particular how normative singlehood is at a given time. There are two opposing perspectives in this regard. According to the sociocultural norm perspective (Eck & Gebauer, 2022), personality effects (i.e., the effect of personality on satisfaction) are stronger when the outcomes are socioculturally more (vs. less) normative. This is because certain personality traits encourage assimilation to sociocultural norms (e.g., extraverts gain positive social attention from meeting sociocultural norms). The paradoxical theory of personality coherence (Caspi & Moffitt, 1993; Neyer et al., 2014), on the other hand, posits that personality effects are stronger in less (vs. more) normative contexts because less prescriptive behavioral rules allow people to express their personality to a greater extent. Applied to the present study, the first perspective would suggest stronger personality effects on satisfaction in later-born cohorts (where singlehood is more normative), while the second perspective would suggest stronger personality effects in earlier-born cohorts (where singlehood is less normative).

## The Present Study

The goal of this preregistered study was to examine whether satisfaction of singles in their adolescence, emerging adulthood, and established adulthood has changed over historical time. We examined life satisfaction as a general indicator of well-being alongside singlehood satisfaction, a domain-specific measure. By analyzing these indicators separately, we investigated if cohort-related effects affected not only singlehood satisfaction but also the overall satisfaction of singles. Moreover, we tested the moderating effects of individual factors including age, gender, extraversion, and neuroticism. We used representative data from a German cohort-sequential study, including four cohorts (born between 1971 and 2003) who annually provided data on their satisfaction between the time periods 2008 to 2011 and 2018 to 2021. This design allowed us to compare earlier-born and later-born singles during adolescence (14-20 years), emerging adulthood (24-30 years), and established adulthood (34-40 years), thereby separating age-related effects from cohort-related effects.

We expected a higher prevalence of singles in the later-born (vs. earlier-born) cohort (Hypothesis 1) and that the higher prevalence is related to higher satisfaction (i.e., satisfaction with singlehood and life satisfaction; Hypothesis 2). Consequently, we expected that later-born (vs. earlier-born) singles would report higher satisfaction (Hypothesis 3). Regarding individual factors, we hypothesized that younger (vs. older) singles would report higher satisfaction (Hypothesis 4) and that female (vs. male) adult singles would report lower satisfaction (Hypothesis 5). We also expected age (Hypothesis 6) and gender (Hypothesis 7) effects to be stronger among earlier-born (vs. later-born) singles. Moreover, we anticipated higher satisfaction among singles with higher (vs. lower) extraversion (Hypothesis 8) and lower (vs. higher) neuroticism (Hypothesis 9). We explored whether the effects of extraversion (Research Question 1) and neuroticism (Research Question 2) varied between later-born and earlier-born cohorts. Finally, we anticipated a decline in satisfaction over the time of being single (Hypothesis 10).

## Method

The hypotheses, design, and data analyses were preregistered at the Open Science Framework (OSF; https://osf.io/8xf95/). Any deviations from the preregistered plan are reported in Supplemental Table S1. Codebook and analysis code are available on the OSF (https://osf.io/pdcg9/).

We used data from the Panel Analysis of Intimate Relationships and Family Dynamics (*pairfam*, Release 13.0, Brüderl et al., 2022), a multi-disciplinary, longitudinal study on romantic relationship and family dynamics in Germany.^1^ Data were assessed annually through computer-assisted personal interviews (CAPI) and computer-assisted self-interviews (CASI). Started in 2008, *pairfam* currently consists of 13 waves of data collected from participants selected through stratified random sampling from population registers. The target population included all German-speaking individuals living in private households in Germany who were born within the years 1991 to 1993, 1981 to 1983, and 1971 to 1973. The initial sample at Wave 1 included 12,402 participants. At Wave 11, a refreshment sample was introduced, including a new birth cohort (2001-2003), with the sampling procedure remaining consistent with that of the original sample. Compliance with ethical standards for German social research and data protection laws was followed throughout data preparation and collection (German Research Foundation, Register Number NE633/10–3).

### Participants

**Figure 1.**
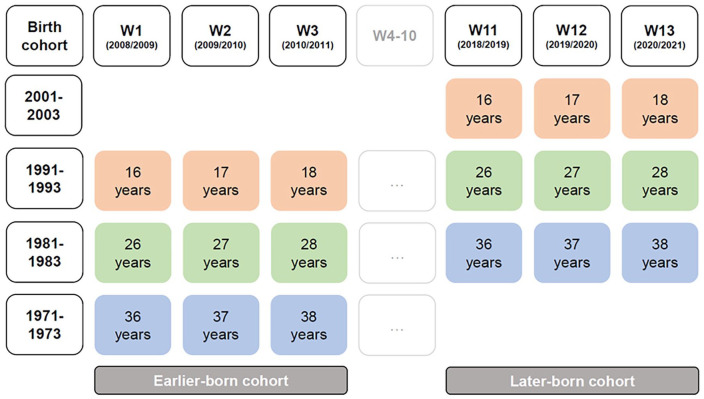
Overview of the Cohort-Sequential Study Design. *Note*. Four birth cohorts (first column) provided data on three consecutive assessments (W1-3 and/or W11-13), allowing for cohort comparisons between the earlier-born cohort and later-born cohort within three age groups: adolescence (orange), emerging adulthood (green), and established adulthood (blue). The years correspond to the average age at each assessment.

We used data from Waves 1 to 3 and from Waves 11 to 13 in our cohort-sequential study design and refer to these waves as T1 (Waves 1 and 11), T2 (Waves 2 and 12), and T3 (Waves 3 and 13), depending on the cohort under investigation (see Figure 1). The focus on these waves was crucial to disentangle age effects (i.e., changes in satisfaction due to individuals’ age) from cohort effects (i.e., differences in satisfaction due to different birth years; Bühler & Nikitin, 2020). Participants belonged to four birth cohorts and three age groups. Specifically, participants were born between 1971 and 1973, 1981 and 1983, 1991 and 1993, and 2001 and 2003. Mean ages during the study period (T1-T3) ranged from 16 to 18 years (adolescents), 26 to 28 years (emerging adults), and 36 to 38 years (established adults). As illustrated in Figure 1, the cohort-sequential design allowed for cohort comparisons within each age group. Specifically, we labeled individuals from birth cohorts 1991 to 1993, 1981 to 1983, and 1971 to 1973 who participated in Waves 1 to 3 as *earlier-born cohort* and individuals from birth cohorts 2001 to 2003, 1991 to 1993, and 1981 to 1983 who participated in Waves 11 to 13 as *later-born cohort*. This procedure allowed us to compare, for example, earlier-born adolescents (orange in the left part of Figure 1) to later-born adolescents (orange in the right part of Figure 1).^2^

To test the prevalence hypotheses (Hypotheses 1 and 2), we used a sample of 17,697 individuals from Wave 1 (2008-2009) as the earlier-born cohort, and from Wave 11 (2018-2019) as the later-born cohort, who provided information on their relationship status.^3^ The earlier-born cohort included 12,358 (*M* = 25.9 years old, 51.4% female) and the later-born cohort included 5,339 (*M* = 24.7 years old, 52.4% female) participants. Please see Table 1 for more information regarding the proportion of singles in each cohort.

**Table 1.** Descriptive Information of the Study Sample Separated by Cohort and Age Group.

Variable	Overall	Adolescents	Emerging adults	Established adults
*Earlier-born cohort*				
Single individuals/cohort	5,124/12,358	3,195/4,320	1,234/3,990	695/4,048
Sample size	1,774	1,137	364	273
Percentage female	39	38	35	49
Age, *M* (*SD*)	21.10 (7.65)	15.88 (0.87)	26.09 (0.88)	36.21 (0.88)
Singlehood satisfaction, *M* (*SD*)	6.64 (2.33)	6.93 (2.26)	6.14 (2.32)	6.12 (2.47)
Life satisfaction, *M* (*SD*)	7.49 (1.81)	7.94 (1.49)	6.80 (1.90)	6.51 (2.17)
Extraversion, *M* (*SD*)	3.36 (0.85)	3.40 (0.86)	3.26 (0.81)	3.31 (0.87)
Neuroticism, *M* (*SD*)	2.66 (0.81)	2.62 (0.80)	2.69 (0.82)	2.77 (0.81)
*Later-born cohort*				
Single individuals/cohort	2,529/5,339	1,895/2,476	368/1,209	266/1,654
Sample size	1,162	790	192	180
Percentage female	46	45	46	48
Age, *M* (*SD*)	20.88 (7.54)	16.12 (0.90)	26.21 (0.97)	36.08 (0.90)
Singlehood satisfaction, *M* (*SD*)	6.98 (2.45)	7.46 (2.23)	6.16 (2.43)	5.73 (2.72)
Life satisfaction, *M* (*SD*)	7.65 (1.75)	8.04 (1.52)	6.94 (1.94)	6.65 (1.81)
Extraversion, *M* (*SD*)	3.24 (0.91)	3.21 (0.90)	3.35 (0.95)	3.23 (0.90)
Neuroticism, *M* (*SD*)	2.87 (0.84)	2.85 (0.84)	2.89 (0.85)	2.92 (0.86)

*Note*. Single individuals/cohort describes the proportion of single individuals in each cohort at T1 (Wave 1 for earlier-born cohort and Wave 11 for later-born cohort). Age, singlehood satisfaction, and life satisfaction refer to T1. Extraversion and neuroticism were assessed in Waves 2 (for earlier-born cohort), 10 (for later-born emerging and established adults), and 11 (for later-born adolescents). Satisfaction with singlehood and life satisfaction ranged from 0 to 10. Extraversion and neuroticism ranged from 1 to 5.

For the remaining hypotheses (Hypotheses 3-10) and research questions (Research Questions 1 and 2), we focused on participants who were continuously single between T1 and T3 and therefore excluded participants who reported being in a romantic relationship at some point during this period (“Do you currently have a partner?” “Did you have a partner sometime after [date of interview previous wave]?”; *n* = 14,761). This was done to account for and illustrate changes in satisfaction over time of being single. The selected sample consisted of 2,936 individuals (*M* = 21.01 years old, *SD* = 7.60 years) who were single throughout the study period and reported on either their satisfaction with singlehood at T1, their life satisfaction at T1, or both. Table 1 shows the descriptive information of the study sample separated by cohort and age group.

An a-priori power analysis based on Monte Carlo simulations (1,000 simulations, .80% power, intraclass correlation coefficient [ICC] = .50, α = .01, two-tailed test) using the package *SIMR* (Green & MacLeod, 2016) in R (R Core Team, 2020) suggested that the sample size was sufficiently large (*N* > 1,600) to detect small (*b* = .10) Level 1 (time) and Level 2 (e.g., cohort) main effects as well as medium (*b* = .30) cross-level interaction effects (e.g., time × age).

### Measures

#### Satisfaction With Singlehood

Satisfaction with singlehood was measured at each assessment with a single item: “How satisfied are you with your situation as a single?,” ranging from 0 (*very dissatisfied*) to 10 (*very satisfied*). This item was assessed in the CAPI mode and presented exclusively to people who have been single for a minimum of 3 months (98% of the sample at T1).

#### Life Satisfaction

Life satisfaction was measured at each assessment with one item: “All in all, how satisfied are you with your life at the moment?” The item was assessed in the CAPI mode ranging from 0 (*very dissatisfied*) to 10 (*very satisfied*).

#### Personality Traits

Personality traits were assessed in Waves 2, 10, and 11 (the latter only for the refreshment sample) in the CASI mode with a short version of the Big Five Inventory (Rammstedt & John, 2005). The dimensions extraversion (e.g., “I get enthusiastic easily and can motivate others easily”) and neuroticism (e.g., “I worry a lot”) were assessed with four items each. The response format ranged from 1 (*absolutely incorrect*) to 5 (*absolutely correct*). For each trait, we used wave-specific mean scores. Specifically, for individuals in the earlier-born cohort, we used personality-trait items from Wave 2; for individuals in the later-born cohort, we used personality-trait items from Waves 10 (birth cohorts 1991-1993 and 1981-1983) and 11 (birth cohort 2001-2003). Thus, participants varied in the timing of their personality-trait assessments. Despite potential changes over time, traits generally maintain moderate to strong rank-order stability over periods ranging from 4 to 12 years, suggesting enduring stability within shorter time frames (Seifert et al., 2022).

We also controlled for the effects of the remaining traits, namely agreeableness (four items; e.g., “I trust others easily and believe that people are inherently good”), conscientiousness (four items; e.g., “I complete my tasks thoroughly”), and openness (five items; e.g., “I am very imaginative”) in additional analyses.

#### Age and Gender

At T1, participants provided information on their age and gender (0 = *male*, 1 = *female*) in the CAPI mode.

### Data Analyses

#### Preparation

To prepare the data for the analyses, we created two additional variables: intraindividual change over time and prevalence of singles. First, intraindividual change describes the number of years since baseline (i.e., T1), ranging from 0 (T1) to 2 (T3). This variable was necessary to examine change in singles’ satisfaction over the time of being single.

Second, we calculated the proportion of singles at T1 (Wave 1 for earlier-born cohort and Wave 11 for later-born cohort), which was done with the prevalence sample of *N* = 17,697 participants. Higher values indicated a greater share of singles at T1 in the respective cohort.

#### Analytic Strategy

We tested differences between the later-born and earlier-born cohort in the prevalence of singles (Hypothesis 1) using a chi-square test and computed Spearman’s rank correlations to assess the association between the prevalence of singles and satisfaction at T1 (Hypothesis 2). The remaining hypotheses (Hypotheses 3-10) were all tested using multilevel growth-curve models.

We examined four models for satisfaction with singlehood and four models for life satisfaction. We note that the sequence of these models does not follow the sequence of the hypotheses because models were constructed in a similar stepwise manner as in previous studies (e.g., Brandt et al., 2022), and the relevant main effects and interaction terms were included in different models. In Model 1, we tested intraindividual change in singles’ satisfaction (Hypothesis 10) and examined age-related differences (Hypothesis 4). At Level 1, participants’ satisfaction with singlehood (or life satisfaction) was predicted from the person-specific intercept, person-specific linear slope, and time-specific residual score. At Level 2, participants’ age group was added as a predictor and as a cross-level interaction with time. In Model 2, we tested cohort-related differences (Hypothesis 3) and age–cohort interaction effects (Hypothesis 6). We did so by extending Model 1 by including participants’ cohort membership as an additional predictor at Level 2 as well as interaction terms with time (cross-level) and age group (same-level). In Model 3, we tested gender-related differences (Hypothesis 5) and gender–cohort interaction effects (Hypothesis 7). We did so by extending Model 2 by including participants’ gender as an additional predictor at Level 2 as well as interaction terms with time (cross-level), age group, and cohort membership (both same-level). In Model 4, we tested the effects of extraversion and neuroticism on singles’ satisfaction (Hypotheses 8 and 9) and personality–cohort interaction effects (Research Questions 1 and 2). Similar to Model 3, we extended Model 2 by including participants’ extraversion (or neuroticism, respectively) at Level 2 as well as their interaction terms with time (cross-level), age group, and cohort membership (both same-level). After computing the models for both traits separately, we included all Big Five traits simultaneously to control for variance shared by all five traits. A summary of the models that were used to test the hypotheses is shown in Table 3 (first two columns).

As preregistered, we reran all models with participants’ gender as an additional control variable (expect for Model 3, in which gender was already included). Given the number of statistical models, we evaluated significance of the multilevel growth-curve models at α level of .01 to address potentially inflated Type 1 error rates. We only interpreted coefficients that were significant and showed effects in the same direction in the models with and without controlling for gender. To get a sense of the magnitude of effects, we determined the proportionate decrease in residual variance of satisfaction from the model including the given effect to the model without the given effect. This indicates the amount of variance accounted for by the specific effect, in comparison to the amount of variance in the outcome that remains unexplained (Lorah, 2018). All models were estimated in R (R Core Team, 2020) using the *lme4* package (Bates et al., 2015).

## Results

### Descriptive Results

Table 2 shows the means, standard deviations, and zero-order correlations among the study variables across birth cohorts and age groups (see Supplemental Table S2 for means and standard deviations separated by birth cohorts and age groups). Satisfaction with singlehood and life satisfaction were positively correlated with each other: The more satisfied singles felt with their singlehood, the more satisfied they were with their lives. Both satisfaction measures were positively associated with extraversion and negatively associated with neuroticism.

**Table 2.** Descriptive Characteristics and Pearson Correlations Among the Study Variables.

Variable	1	2	3	4	5	6	7	8
1. T1 singlehood satisfaction (*n* = 2,883)								
2. T2 singlehood satisfaction (*n* = 2,894)	.48							
3. T3 singlehood satisfaction (*n* = 2,890)	.43	.55						
4. T1 life satisfaction (*n* = 2,935)	.34	.25	.24					
5. T2 life satisfaction (*n* = 2,932)	.26	.32	.23	.54				
6. T3 life satisfaction (*n* = 2,934)	.23	.23	.30	.45	.53			
7. Extraversion (*n* = 2,927)	.07	.05	.07	.16	.20	.12		
8. Neuroticism (*n* = 2,928)	−.16	−.15	−.16	−.30	−.33	−.28	−.27	
*M*	6.77	6.51	6.48	7.55	7.49	7.28	3.31	2.74
*SD*	2.39	2.48	2.39	1.78	1.73	1.73	0.88	0.83

*Note*. Satisfaction with singlehood and life satisfaction ranged from 0 to 10. Extraversion and neuroticism ranged from 1 to 5. Extraversion and neuroticism were assessed in Waves 2, 6, and 11, depending on participants’ cohort membership. All correlations were significant at *p* < .005.

### Prevalence of Singles

First, we inspected the prevalence of singles in each cohort (Hypothesis 1) and its association with satisfaction (Hypothesis 2). An overview of the main findings for each hypothesis is shown in Table 3. The chi-square test indicated that the later-born (47%) and earlier-born (42%) cohorts significantly differed in their proportions of singles (*χ*^2^(1) = 52.73, *p* < .001). Thus, in line with Hypothesis 1, people from the later-born cohort were more often single in 2018-2019 than people from the earlier-born cohort in 2008-2009.

**Table 3.** Summary of the Main Findings.

Hypothesis	Analysis method	Main finding	Hypothesis supported?
Prevalence of singles (Hypothesis 1)	Chi-squared test	Higher prevalence of singles among adolescents born in 2001 to 2003 (vs. 1991-1993)	Partially supported (for adolescents)
Correlation between prevalence of singles and satisfaction (Hypothesis 2)	Spearman’s correlation	Higher prevalence of singles related to higher singlehood satisfaction and higher life satisfaction across all age groups	Supported
Cohort membership (Hypothesis 3)	Model 2	Higher singlehood satisfaction among adolescents born in 2001 to 2003 (vs. 1991-1993; no differences in other age groups and in life satisfaction)	Partially supported (for adolescents and singlehood satisfaction)
Age (Hypothesis 4)	Model 1	Higher singlehood satisfaction and higher life satisfaction among adolescents (vs. adults). Higher life satisfaction but not higher singlehood satisfaction among emerging (vs. established) adults	Supported for life satisfaction and partially supported for singlehood satisfaction
Gender (Hypothesis 5)	Model 3	Higher singlehood satisfaction among female (vs. male) established adults (no gender differences in other age groups and in life satisfaction)	Not supported
Age–cohort interaction (Hypothesis 6)	Model 2	Higher singlehood satisfaction among adolescents born in 2001 to 2003 (vs. 1991-1993; no cohort differences in other age groups)	Not supported
Gender–cohort interaction (Hypothesis 7)	Model 3	Gender differences more pronounced among later-born (vs. earlier-born) cohort	Not supported
Extraversion (Hypothesis 8)	Model 4	Extraversion related to higher life satisfaction (but not to singlehood satisfaction)	Partially supported (for life satisfaction)
Neuroticism (Hypothesis 9)	Model 4	Neuroticism related to both lower singlehood satisfaction and lower life satisfaction	Supported
Intraindividual change (Hypothesis 10)	Model 1	Decline in singlehood satisfaction across all age groups (but decline in life satisfaction only for adolescents)	Partially supported (for singlehood satisfaction)
Extraversion–cohort interaction (Research Question 1)	Model 4	Weaker effect of extraversion on life satisfaction in later-born cohort (but not on singlehood satisfaction)	—
Neuroticism–cohort interaction (Research Question 2)	Model 4	Effects of neuroticism on singlehood satisfaction and life satisfaction independent of cohort membership	—

*Note*. “—” indicates non-applicability due to the absence of formulated hypotheses. Model 1 = satisfaction predicted by person-specific intercept, slope, and residual (Level 1) and age (Level 2); Model 2 = cohort membership (Level 2) as additional predictor; Model 3 = gender (Level 2) as additional predictor; Model 4 = extraversion (or neuroticism, respectively) as additional predictor.

However, as the proportion of singles greatly differed between the age groups (17% in established adulthood, 31% in emerging adulthood, and 75% in adolescence) and participants of the later-born cohort were, on average, significantly younger (*M* = 24.73, *SD* = 8.69) than participants of the earlier-born cohort (*M* = 25.87, *SD* = 8.34, *t*(9,768) = −8.10, *p* < .001), we conducted non-preregistered supplemental analyses and tested Hypothesis 1 also within age groups. We observed significant cohort-related differences for adolescents: Adolescents born in 2001 to 2003 were significantly more often single (77%) than adolescents born in 1991 to 1993 (74%; *χ*^2^(1) = 5.42, *p* = .020). The proportion of singles did neither differ between emerging adults born in 1981 to 1983 (31%) and 1991 to 1993 (30%) nor differ between established adults born in 1981 to 1983 (16%) and 1971 to 1973 (17%), both *p*s ≥ .339.

The proportion of singles was significantly associated with higher singlehood satisfaction (*r*s(7,488) = .20, *p* < .001) and higher life satisfaction (*r*s(7,637) = .33, *p* < .001). Thus, in line with Hypothesis 2, the higher the share of singles in a birth cohort, the more satisfied singles were with their singlehood and life in general.

### Intraindividual Change and Age Effects

In Model 1, we assessed intraindividual change (Hypothesis 10) and age-related differences (Hypothesis 4) in satisfaction. The full results of Model 1 are shown in Supplemental Table S3.

#### Singlehood Satisfaction

Singles decreased in their satisfaction with singlehood (*b* = −0.17, 95% confidence interval, CI [−0.23, −0.11], *p* < .001) over the time of being single. This was the case for all age groups, as we found no significant interaction between age group and time. Emerging (*b* = −1.07, 95% CI [−1.29, −0.86], *p* < .001) and established (*b* = −1.21, 95% CI [−1.44, −0.98], *p* < .001) adults were, on average, less satisfied with being single at T1 than adolescents. Comparisons within adulthood indicated that established adults, compared to emerging adults, were similarly satisfied with their singlehood at T1 (*b* = −0.14, 95% CI [−0.42, 0.14], *p* = .337). Controlling for gender did neither change the direction nor change the significance of the effects (see Supplemental Table S3).

#### Life Satisfaction

Singles decreased in their life satisfaction (*b* = −0.19, 95% CI [−0.23, −0.15], *p* < .001) over the time of being single. However, the decrease was only significant for adolescents but not emerging (*b* = −0.07, 95% CI [−0.15, 0.01], *p* = .072) or established (*b* = 0.03, 95% CI [−0.05, 0.12], *p* = .421) adults. Emerging (*b* = −1.10, 95% CI [−1.25, −0.94], *p* < .001) and established (*b* = −1.42, 95% CI [−1.59, −1.25], *p* < .001) adults were, on average, less satisfied with their life compared to adolescents at T1. Comparisons within adulthood indicated that established adults, compared to emerging adults, reported significantly lower life satisfaction at T1 (*b* = −0.32, 95% CI [−0.53, −0.12], *p* = .002). Controlling for gender did neither change the direction nor change the significance of the effects (see Supplemental Table S3).

In sum, the findings indicate a significant decrease in singlehood satisfaction across age groups, while a decrease in life satisfaction was observed only among adolescents, partially supporting Hypothesis 10. Conversely, although life satisfaction decreased with each age group, singlehood satisfaction was highest in adolescence, with no differences between emerging and established adults, thus partially supporting Hypothesis 4.

### Cohort Effects

In Model 2, we tested cohort-related differences in satisfaction (Hypothesis 3) as well as age–cohort interactions (Hypothesis 6). The full results are shown in Supplemental Table S4.

#### Singlehood Satisfaction

**Figure 2.**
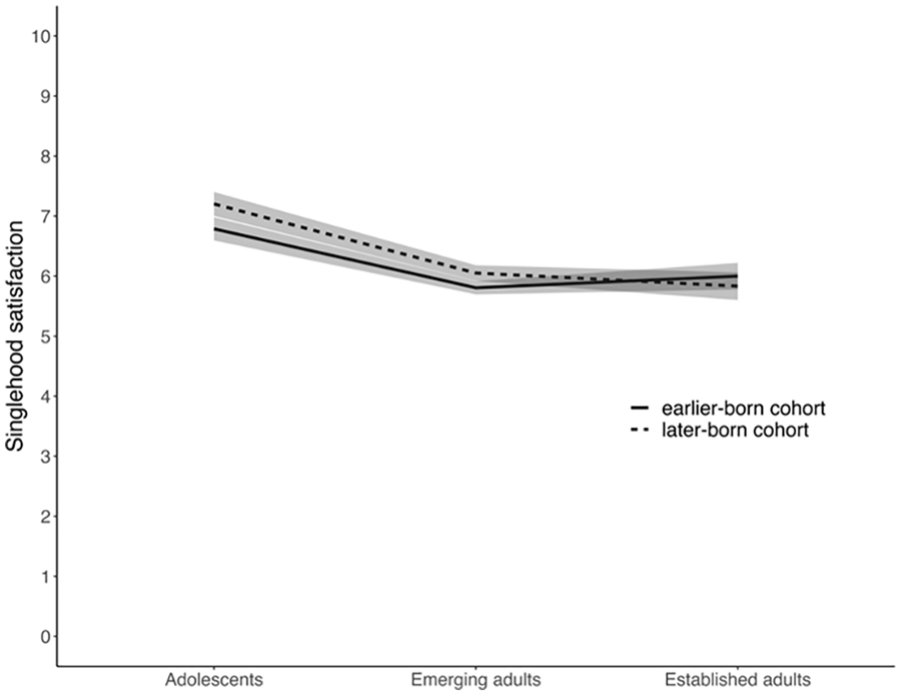
Interaction Effect Between Age Group and Cohort Membership on Singlehood Satisfaction. *Note*. Shaded areas represent 95% confidence intervals.

Later-born singles, compared to earlier-born singles, reported higher singlehood satisfaction at T1 (2018-2019 for later-born singles and 2008-2009 for earlier-born singles, respectively; *b* = 0.43, 95% CI [0.23, 0.62], *p* < .001). There were no cohort differences in the intraindividual change over time, indicating similar trajectories in singlehood satisfaction for both cohorts. Controlling for gender did not change the direction or significance of the effects. There were significant interaction effects between age group and cohort membership on singlehood satisfaction at T1. Figure 2 illustrates the interaction effect on singlehood satisfaction (controlling for gender), showing more pronounced age differences in the later-born cohort than in the earlier-born cohort. The interaction plot also shows that only later-born adolescents, but not later-born emerging or established adults, reported significantly higher singlehood satisfaction than their earlier-born counterparts.

#### Life Satisfaction

Later-born singles did not differ in their life satisfaction (*b* = −0.04, 95% CI [−1.17, 0.10], *p* = .620) from earlier-born singles at T1 but differed in their intraindividual change over time. Specifically, there was a stronger decline in life satisfaction between T1 and T3 for the later-born cohort (i.e., between 2018-2019 and 2020-2021) compared to the earlier-born cohort (i.e., between 2008-2009 and 2010-2011; *b* = −0.14, 95% CI [−0.21, −0.07], *p* < .001). Again, controlling for gender did not change the direction or significance of the effects.

In sum, the findings partially support Hypothesis 3, showing that adolescents born in 2001 to 2003 reported greater singlehood satisfaction than those born in 1991 to 1993. No differences were found among adults for life satisfaction at the initial assessment, although later-born cohort declined more steeply in life satisfaction over time. Contrary to Hypothesis 6, the results suggested more pronounced age differences among later-born singles compared to their earlier-born counterparts.

### Gender Effects

In Model 3, we examined gender differences in satisfaction (Hypothesis 5) as well as gender–cohort interactions (Hypothesis 7). The full results are reported in Supplemental Table S5.

#### Singlehood Satisfaction

Female emerging adults, compared to male emerging adults, did not differ in their singlehood satisfaction at T1 (*b* = 0.36, 95% CI [0.02, 0.71], *p* = .041). However, female established adults, compared to male established adults, reported significantly higher singlehood satisfaction at T1 (*b* = 0.65, 95% CI [0.28, 1.02], *p* = .001). There was a significant interaction effect of gender and cohort on singlehood satisfaction (*b* = 0.42, 95% CI [0.14, 0.71], *p* = .003) suggesting that gender differences were more pronounced among later-born (vs. earlier-born) singles. No gender differences were found in the intraindividual change.

#### Life Satisfaction

There were no gender differences in life satisfaction at T1 among emerging adults (*b* = −0.01, 95% CI [−0.26, 0.24], *p* = .943) nor established adults (*b* = 0.13, 95% CI [−0.13, 0.40], *p* = .330). We found no significant interaction effect of gender and cohort on life satisfaction at T1 (*b* = −0.12, 95% CI [−0.31, 0.08], *p* = .257). Again, there were no gender differences in intraindividual change.

In sum, our findings suggested higher singlehood satisfaction among female established adults compared to their male counterparts, thereby not supporting Hypothesis 5. Contrary to Hypothesis 7, the interaction effect between gender and cohort was either non-significant or in the opposite direction to what we expected.

### Personality Effects

In Model 4, we tested the effects of extraversion (Hypothesis 8) and neuroticism (Hypothesis 9) on satisfaction. In addition, we explored whether the effects of extraversion (Research Question 1) and neuroticism (Research Question 2) on satisfaction were stronger in later-born or earlier-born cohort. The full results are shown in Supplemental Tables S6 and S7, respectively. The results including all Big Five personality traits are shown in Supplemental Table S8.

#### Singlehood Satisfaction

Extraversion was positively related to singlehood satisfaction at T1 (*b* = 0.23, 95% CI [0.12, 0.35], *p* < .001). Inclusion of gender as a covariate did neither change the direction nor change the significance of the effects. However, the simultaneous inclusion of all Big Five traits in one model resulted in a non-significant effect of extraversion on singlehood satisfaction at T1 (see Supplemental Table S8). Effects of extraversion on singlehood satisfaction were unrelated to cohort membership, as we found no significant interaction effect (*b* = −0.17, 95% CI [−0.33, −0.01], *p* = .038). Similarly, no effects of extraversion were found on the intraindividual change.

Neuroticism was negatively related to singlehood satisfaction at T1 (*b* = −0.56, 95% CI [−0.68, −0.44], *p* < .001). Inclusion of gender and inclusion of all Big Five traits simultaneously in one model did not change the direction or the significance of the effects. The effect of neuroticism on singles’ satisfaction was weaker among later-born singles compared to earlier-born singles at T1 (*b* = 0.24, 95% CI [0.07, 0.41], *p* = .005), but the effect was not replicated after including all Big Five traits. Again, there were no effects of neuroticism on the intraindividual change.

#### Life Satisfaction

Extraversion was positively related to life satisfaction at T1 (*b* = 0.43, 95% CI [0.35, 0.51], *p* < .001). Inclusion of gender and inclusion of all Big Five traits did not change the direction or the significance of the effects. The effect of extraversion on life satisfaction was weaker among later-born singles, as we found a significant interaction effect (*b* = −0.18, 95% CI [−0.29, −0.07], *p* = .001). No effects of extraversion were found on intraindividual change.

Neuroticism was negatively related to life satisfaction at T1 (*b* = −0.70, 95% CI [−0.78, −0.62], *p* < .001). Inclusion of gender and inclusion of all Big Five traits did not change the direction or the significance of the effects. Effects of neuroticism on life satisfaction were unrelated to cohort membership, as we found no significant interaction effect (*b* = 0.11, 95% CI [−0.01, 0.22], *p* = .056). No effects of neuroticism were found on the intraindividual change in life satisfaction.

In sum, Hypothesis 8 was partially supported, as the results indicated a robust positive association between extraversion and life satisfaction, but not between extraversion and singlehood satisfaction. In addition, neuroticism was related to both lower singlehood satisfaction and lower life satisfaction, fully supporting Hypothesis 9. While the effects of neuroticism on satisfaction remained largely consistent across cohorts, the effect of extraversion on life satisfaction appeared weaker among later-born cohorts.

### Overview of Effect Sizes

Overall, the effects of cohort membership were rather small accounting for 1.7% of the variance in singlehood satisfaction and accounting for 0.1% of the variance in life satisfaction. Larger effects were found for age and neuroticism, but these effect sizes were still small to medium. Specifically, age accounted for 9.7% and 18.8% of the variances in singlehood satisfaction and life satisfaction, respectively. Neuroticism accounted for 5.4% and 19% of the variances in singlehood satisfaction and life satisfaction, respectively. Small effect sizes were found for gender (2.9% and 0.9% of the variances in singlehood satisfaction and life satisfaction, respectively) and extraversion (1.0% and 6.3% of the variances in singlehood satisfaction and life satisfaction, respectively).

### Supplementary Analyses

Based on valuable feedback we received during the review process, we conducted two additional analyses regarding the cohort effects. First, we tested potential mechanisms that might account for the historical increase found among adolescents’ singlehood satisfaction (see p. 12-13 in the Supplemental Material for details). Second, we tested whether findings concerning the cohort effects were unique to the context of singlehood or whether partnered individuals also have experienced historical changes in their satisfaction with their relationship and their life (see p. 14-16 in the Supplemental Material for details).

We explored three factors potentially contributing to adolescents’ increased satisfaction with singlehood: *desire for a romantic relationship*, *sexual satisfaction*, and *friendship satisfaction*. Singles with lower romantic desire, higher sexual satisfaction, and greater friendship satisfaction tend to be more satisfied (Girme et al., 2022) and changes in these factors may explain the rise in singlehood satisfaction among adolescents. Our results indicated that adolescents born in 2001 to 2003 showed lower romantic desire compared to those born in 1991 to 1993. As adolescents with a higher desire for a romantic relationship tended to be less satisfied with being single, the overall decline in romantic desire may have benefited adolescents’ satisfaction. Neither sexual satisfaction nor friendship satisfaction contributed to the trend toward increased singlehood satisfaction among adolescents.

We tested whether partnered individuals experienced similar historical changes in their satisfaction compared to single individuals. A total of 5,181 participants who remained in the same romantic relationship throughout the study period were included in the analyses. Results revealed no historical changes in neither relationship satisfaction nor life satisfaction among partnered individuals. However, when examining different age groups within adulthood, a significant interaction effect emerged between age group and cohort membership regarding relationship satisfaction. Specifically, only later-born established adults reported lower relationship satisfaction compared to their earlier-born counterparts, with no such trends observed among later-born adolescents or emerging adults. However, it is important to note that a recent meta-analysis (Bühler et al., 2021) found no cohort effects on relationship satisfaction (mean age = 34.81 years), cautioning against overinterpreting this finding.

## Discussion

Singlehood has significantly changed across many Western industrialized countries, becoming more normative and socially accepted over the last decades (Bühler & Nikitin, 2020; Lesthaeghe, 2014; Tillman et al., 2019). However, whether singles have also become more satisfied over time is still unclear. Using a cohort-sequential design from a large and representative longitudinal study from Germany, we tested whether singles nowadays are more satisfied with their singlehood and life in general compared to previous generations. We observed historical changes for adolescents, but not for emerging and established adults. Specifically, adolescents born in 2001 to 2003 were more often single and more satisfied with being single than adolescents born 10 years earlier. However, the effect sizes were small and individual factors such as age and neuroticism emerged as stronger predictors of singles’ satisfaction than cohort membership.

### Adolescent Singles Are More Satisfied Today

The norms surrounding romantic relationships and singlehood underwent significant changes in Western societies (Bühler & Nikitin, 2020). People nowadays put more emphasis on individualism and personal autonomy (Lesthaeghe, 2014) and have become more accepting of diverse relationship dynamics including prolonged singlehood (Tillman et al., 2019). These societal changes may have contributed to higher satisfaction among singles today.

We found historical changes among adolescents in Germany, with adolescents born in 2001 to 2003 showing a 3% greater chance of being single compared to adolescents born 10 years earlier, specifically in 1991 to 1993. This rise in proportion of singles corresponded to increased satisfaction with singlehood among later-born adolescents. The historical increase in satisfaction appears specific to the context of singlehood, as we found no evidence for a general increase in neither relationship satisfaction nor overall life satisfaction. That adolescents are more satisfied with being single today aligns with our expectations as well as with previous research showing a reduced emphasis on romantic partners for personal happiness in today’s adolescents (Scheling & Richter, 2021). Compared to adolescents born a decade earlier, those born in 2001 to 2003 also reported a reduced desire for a romantic partner, as indicated by our supplementary analyses. Given that a lower desire for a romantic partner is positively related to singles’ well-being (Kislev, 2021), this decline in romantic desire may contribute to the observed increase in singlehood satisfaction over time. Specifically, adolescents nowadays may postpone entering relationships, prioritize personal autonomy and individual fulfillment over romantic involvement, and embrace singlehood more openly. However, these explanations are speculative and warrant further investigation.

We anticipated that this historical increase in singles’ satisfaction would also manifest among emerging and established adults. Again, we can only speculate about the reasons why historical changes were not observable among emerging and established adult singles. Adolescents may exhibit a heightened responsiveness to sociohistorical changes, given that this age period involves increased susceptibility to external influences. Individuals start navigating through a broader range of social contexts and experiences beyond the family context (Steinberg & Morris, 2001), and place significant importance on peer acceptance and belonging (van de Bongardt et al., 2015), while actively exploring and shaping their identities through new social experiences (Bogaerts et al., 2019). Moreover, their pervasive use of social media exposes them to a wide array of romantic relationship options, making them more receptive to evolving sociocultural messages (Vaterlaus et al., 2018). Thus, changing norms may have a greater impact on adolescents’ dating behavior and singlehood satisfaction.

Together, the findings support lifespan perspectives (Baltes et al., 2007; Bronfenbrenner, 1986; Schaie, 1965) showing that experiences of singlehood are not static but subject to both historical and age-related influences. Future research delving into individuals’ perceptions of societal pressures and sociocultural norms regarding their romantic experiences could provide valuable insights into the mechanisms behind this age-specific historical rise in satisfaction.

### Younger Age and Lower Neuroticism Contribute to Higher Satisfaction

Singlehood experiences are diverse and multifaceted and can vary based on individual factors such as demographics and personality traits (Girme et al., 2022). We investigated how age, gender, extraversion, and neuroticism contributed to singles’ satisfaction. Among these factors, age and neuroticism emerged as particularly robust predictors of satisfaction.

Specifically, in line with our hypotheses, adolescent singles were more satisfied with being single and life in general than emerging and established adults. Even though most people living in Germany have their first romantic relationship experiences during adolescence (Gonzalez Avilés et al., 2021), singlehood remains a highly normative experience among adolescents, with approximately 75% of adolescents reporting being single in the current study. Adolescents may perceive their single status as more aligned with the relationship status of their peers, which may contribute to their higher levels of satisfaction (Havighurst, 1972). However, the finding that adolescents were most satisfied not only with being single but also with life in general suggests that the differences in satisfaction might extend beyond singlehood alone. Instead, adolescents’ satisfaction could be influenced by broader and more universal factors tied to age. Emerging and established adults reported similar levels of satisfaction with their singlehood, contrary to our hypothesis. Unlike adolescence, where the majority are single, singlehood appears to be less common among both emerging and established adults, with rates of 31% and 17%, respectively. Thus, the differences within adult age groups may be less pronounced compared to adolescents.

Regarding personality traits, the findings showed that singles with higher levels in neuroticism were less satisfied with being single and life in general. Higher neuroticism is linked to increased susceptibility to negative emotions and less adaptive coping strategies for personal challenges (Heller et al., 2004). This tendency may also affect individuals’ experiences with being single. Specifically, singles exhibiting higher neuroticism may experience heightened distress and persistent worries about their single status, fearing a lack of future romantic connections (Spielmann et al., 2013), potentially undermining their satisfaction.

While the effects of age and neuroticism (partially) aligned with our hypotheses, the effects of extraversion and gender on satisfaction were smaller or non-significant, contrary to our expectations. Specifically, singles with higher extraversion were more satisfied with life, but not with being single. Therefore, while extraversion has been linked to greater life satisfaction and marital satisfaction in the past (Heller et al., 2004), the positive effect does not necessarily apply to the context of singlehood. Rather than extraversion itself, the size and satisfaction with social networks may be more closely related to singles’ satisfaction. Contrary to our expectations, female singles in established adulthood were *more* satisfied with being single than their male counterparts. Women in this life stage may have already passed the average peak of first marriages and childbirth (both around 30 years; Mehta et al., 2020), possibly resulting in less societal pressure and greater satisfaction with being single. Gender differences were stronger in later-born rather than earlier-born cohorts, suggesting that certain sociohistorical changes may have favored female singles more strongly. Future research might benefit from investigating shifts in gender roles as contributing factor to this trend.

We further found some evidence for effects of extraversion (but not neuroticism) depending on the birth cohort. Specifically, extraversion was more strongly related to life satisfaction among earlier-born than later-born singles, which would be in line with the perspective that personality effects are stronger in contexts in which singlehood was less (vs. more) normative (Caspi & Moffitt, 1993; Neyer et al., 2014). However, as this effect was small and not found for singlehood satisfaction, we exercise caution in attributing this effect solely to the context of being single.

In line with our hypothesis and previous research (Oh et al., 2021), singles decreased on average in their singlehood satisfaction over a period of 2 years. This decrease may stem from both individual factors (e.g., changing personal goals) and societal influences (e.g., stigma toward single life) attached to single life (Girme et al., 2022). However, this trend also mirrors the decline observed in other domain-specific satisfactions, such as relationship satisfaction, over time (Bühler et al., 2021) suggesting potential similarities in the factors affecting both singlehood and relationship satisfaction over time. Contrary to our hypothesis, we observed no decline in the life satisfaction of single adults, suggesting that any observed decline may be specific to the context of singlehood.

### Limitations and Outlook

We used a representative and large national sample covering a broad age range (ages 14-40 years), a long study period (2008-2021), and a cohort-sequential design to disentangle age-related influences from cohort-related influences. We furthermore separated global and domain-specific satisfaction and focused exclusively on the experiences among singles, who have received considerably less research attention than partnered individuals (Girme et al., 2022). By doing so, we were able to gain knowledge about the potential factors that might drive singles’ satisfaction.

Despite these strengths, there are some limitations. First, we acknowledge that shifts in societal attitudes may take a considerable amount of time to unfold and affect individuals. It is possible that longer study periods might reveal more pronounced effects of historical changes in singlehood satisfaction. Nonetheless, previous findings (Böger & Huxhold, 2020) indicate significant shifts in singles’ well-being over time periods of 6 years, suggesting that even within shorter time frames, historical changes are observable.

Second, we did not differentiate between period and cohort effects. For example, higher singlehood satisfaction among adolescents born in 2001 to 2003 (vs. 1991-1993) may be due to events taking place between 2018 and 2021 (i.e., period effects) rather than shared experiences during formative years (i.e., cohort effects). Notably, part of the data from the later-born cohort was collected during the COVID-19 pandemic, likely affecting how people evaluate their satisfaction (e.g., Bühler et al., 2023). Although our multilevel analyses showed a stronger decline in life satisfaction between 2018 and 2021 (i.e., later-born cohort) compared to between 2008 and 2011 (i.e., earlier-born cohort), no differences were found in the rate of change in singlehood satisfaction suggesting that the COVID-19 pandemic did not affect individuals’ satisfaction with being single. In addition, cohort differences were interpreted based on their first assessment (2008-2009 for the earlier-born and 2018-2019 for the later-born cohort). Unfortunately, it is not possible to fully disentangling age, period, and cohort effects without ambiguity (Bell, 2020). However, the finding that historical changes were observed only among adolescents, and not among emerging and established adults, suggests that shifts in norms are more likely shaped by the generational characteristics rather than events affecting all individuals at a given time.

Third, we could not test whether the observed effects generalize to age groups older than established adulthood and to cultures outside the Western realm. Although romantic relationships are particularly salient and normative in adolescence and (emerging and established) adulthood, future studies may benefit from examining older singles’ satisfaction over historical time (see Böger & Huxhold, 2020; Park et al., 2022). Similarly, singlehood experiences may differ in countries with more traditional views of marriage and family compared to Germany (Kislev, 2018).

Finally, singles differ on a variety of factors beyond those investigated in the current study, such as attachment style, voluntary singlehood, and attitudes toward marriage and family (Girme et al., 2022). For instance, singles who see their single status as chosen tend to report higher happiness than those who view it as imposed (Adamczyk, 2017). Investigating historical changes in these characteristics and their link to satisfaction is an important avenue for future research.

## Conclusion

Have singles become more satisfied over historical time? Our findings indicate that adolescents born in 2001 to 2003 were more often single and more satisfied with being single than adolescents born a decade earlier. This historical change, however, was not observed among emerging and established adults, pointing to adolescence being a particularly sensitive age period for sociohistorical influences. Across cohorts, individual factors were related to singles’ satisfaction suggesting positive effects of younger age and lower neuroticism. Together, the findings highlight the importance of considering both sociohistorical and individual factors in synergy to arrive at a deeper understanding of singles’ satisfaction.

## Supplemental Material

sj-docx-1-psp-10.1177_01461672241257139 – Supplemental material for Today’s Adolescents Are More Satisfied With Being Single: Findings From a German Cohort-Sequential Study Among 14- to 40-Year-Olds Supplemental material, sj-docx-1-psp-10.1177_01461672241257139 for Today’s Adolescents Are More Satisfied With Being Single: Findings From a German Cohort-Sequential Study Among 14- to 40-Year-Olds by Tita Gonzalez Avilés, Janina Larissa Bühler, Naemi D. Brandt and Franz J. Neyer in Personality and Social Psychology Bulletin

## Notes

1. Data are available at https://www.pairfam.de/.

2. Given that the emerging and established adults of the later-born cohort (Waves 11-13) have been engaged in the study since Wave 1, there is a potential for attrition over time, which could introduce a selection bias. Therefore, we tested whether individuals from the initial *pairfam* sample (*N* = 12,402) who dropped out before Wave 11 (*n* = 8,594) differed from those who remained in the study until Wave 11 (*n* = 3,808) in key study variables at the initial assessment. Results suggested that individuals who participated up to Wave 11 were more likely male (*χ*^2^(1) = 21.81, *p* < .001) and were slightly less satisfied with their single lives (*t*(3,150) = 2.45, *p* = .014, *d* = −0.11) at Wave 1 than those who dropped out. However, there were no significant differences in age at first participation, relationship status, life satisfaction nor in extraversion and neuroticism (all *p*s ≥ .05). Therefore, the results point toward a relatively unbiased sample of participants who remained in the study.

3. For participants who took part in both Waves 1 and 11, we included only their initial reports to reduce potential attrition bias.

## References

[bibr1-01461672241257139] Adamczyk K. (2017). Voluntary and involuntary singlehood and young adults’ mental health: An investigation of mediating role of romantic loneliness. Current Psychology, 36(4), 888–904. 10.1007/s12144-016-9478-3 29200802 PMC5696487

[bibr2-01461672241257139] Arnett J. J. (2000). Emerging adulthood: A theory of development from the late teens through the twenties. American Psychologist, 55(5), 469–480. 10.1037/0003-066X.55.5.469 10842426

[bibr3-01461672241257139] Baltes P. B. Lindenberger U. Staudinger U. M. (2007). Life span theory in developmental psychology. In Damon W. Lerner R. M. (Hrsg.), Handbook of child psychology: Vol. 1. Theoretical models of human development (6th ed., pp. S569–664). John Wiley. 10.1002/9780470147658.chpsy0111

[bibr4-01461672241257139] Barnett R. C. Hyde J. S. (2001). Women, men, work and family: An expansionist theory. American Psychologist, 56(10), 781–796. 10.1037/0003-066X.56.10.781 11675985

[bibr5-01461672241257139] Bates D. Mächler M. Bolker B. Walker S. (2015). Fitting linear mixed-effects models using lme4. Journal of Statistical Software, 67(1), 1–48. 10.18637/jss.v067.i01

[bibr6-01461672241257139] Bell A. (2020). Age period cohort analysis: A review of what we should and shouldn’t do. Annals of Human Biology, 47(2), 208–217. 10.1080/03014460.2019.1707872 32429768

[bibr7-01461672241257139] Bogaerts A. Claes L. Schwartz S. J. Becht A. I. Verschueren M. Gandhi A. Luyckx K. (2019). Identity structure and processes in adolescence: Examining the directionality of between- and within-person associations. Journal of Youth and Adolescence, 48, 891–907. 10.1007/s10964-018-0931-5 30251014

[bibr8-01461672241257139] Böger A. Huxhold O. (2020). The changing relationship between partnership status and loneliness: Effects related to aging and historical time. Journals of Gerontology: Psychological Sciences, 75(7), 1423–1432. 10.1093/geronb/gby153 30590817

[bibr9-01461672241257139] Brandt N. D. Drewelies J. Willis S. L. Schaie K. W. Ram N. Gerstorf D. Wagner J. (2022). Acting like a baby boomer? Birth-cohort differences in adults’ personality trajectories during the last half a century. Psychological Science, 33(3), 382–396. 10.1177/09567976211037971 35192413 PMC9096450

[bibr10-01461672241257139] Bronfenbrenner U. (1986). Ecology of the family as a context for human development: Research perspectives. Developmental Psychology, 22(6), 723–742.

[bibr11-01461672241257139] Brüderl J. Drobnič S. Hank K. Neyer F. J. Walper S. Alt P. . . . Wetzel M. (2022). The German family panel (pairfam). GESIS Data Archive, Cologne, ZA5678. Data file Version 13.0.0. 10.4232/pairfam.5678.13.0.0

[bibr12-01461672241257139] Bühler J. L. Hopwood C. J. Nissen A. Bleidorn W. (2023). Collective stressors affect the psychosocial development of young adults. Social Psychological and Personality Science, 14(6), 708–726. 10.1177/19485506221119018

[bibr13-01461672241257139] Bühler J. L. Krauss S. Orth U. (2021). Development of relationship satisfaction across the life span: A systematic review and meta-analysis. Psychological Bulletin, 10(147), 1012–1053. 10.1037/bul0000342 34928690

[bibr14-01461672241257139] Bühler J. L. Nikitin J. (2020). Sociohistorical context and adult social development: New directions for 21st century research. American Psychologist, 75(4), 457–469. 10.1037/amp0000611 32378942

[bibr15-01461672241257139] Byrne A. Carr D. (2005). Caught in the cultural lag: The stigma of singlehood. Psychological Inquiry, 16(2–3), 84–91. 10.1207/s15327965pli162&3_02

[bibr16-01461672241257139] Caspi A. Moffitt T. E. (1993). When do individual differences matter? A paradoxical theory of personality coherence. Psychological Inquiry, 4(4), 247–271. 10.1207/s15327965pli0404_1

[bibr17-01461672241257139] DePaulo B. Morris W. L. (2005). Singles in society and in science. Psychological Inquiry, 16(2–3), 57–83. 10.1080/1047840X.2005.9682918

[bibr18-01461672241257139] Donnelly K. Twenge J. M. Clark M. A. Shaikh S. K. Beiler-May A. Carter N. T. (2016). Attitudes toward women’s work and family roles in the United States, 1976–2013. Psychology of Women Quarterly, 40(1), 41–54. 10.1177/0361684315590774

[bibr19-01461672241257139] Dupuis H. E. Girme Y. U. (2024). “Cat Ladies” and “Mama’s Boys”: A mixed-methods analysis of the gendered discrimination and stereotypes of single women and single men. Personality and Social Psychology Bulletin, 50, 314–328. 10.1177/01461672231203123 37876182 PMC10860362

[bibr20-01461672241257139] Eck J. Gebauer J. E. (2022). A sociocultural norm perspective on Big Five prediction. Journal of Personality and Social Psychology, 122(3), 554–575. 10.1037/pspp0000387 34516180

[bibr21-01461672241257139] Eckhard J. (2015). Abnehmende Bindungsquoten in Deutschland: Ausmaß und Bedeutung eines historischen Trends [The decrease of couple relationships in Germany: Extent and meaning of a current trend]. Kölner Zeitschrift für Soziologie und Sozialpsychologie, 67(1), 27–55. 10.1007/s11577-014-0296-z

[bibr22-01461672241257139] EuroStat. (2024). Distribution of households by household type from 2003 onwards—EU-SILC survey. http://data.europa.eu/88u/dataset/vq4g2a9iwej8esr1mg

[bibr23-01461672241257139] Fisher A. N. Sakaluk J. K. (2020). Are single people a stigmatized “group”? Evidence from examinations of social identity, entitativity, and perceived responsibility. Journal of Experimental Social Psychology, 86, 103844. 10.1016/j.jesp.2019.103844

[bibr24-01461672241257139] Girme Y. U. Park Y. MacDonald G. (2023). Coping or thriving? Reviewing intrapersonal, interpersonal, and societal factors associated with well-being in singlehood from a within-group perspective. Perspectives on Psychological Science, 18, 1097–1120. 10.1177/17456916221136119 36534959 PMC10475216

[bibr25-01461672241257139] Gonzalez Avilés T. Finn C. Neyer F. J . (2021). Patterns of romantic relationship experiences and psychosocial adjustment from adolescence to young adulthood. Journal of Youth and Adolescence, 50, 550–562. 10.1007/s10964-020-01350-7 33196893 PMC7910227

[bibr26-01461672241257139] Green P. MacLeod C. J. (2016). SIMR: An R package for power analysis of generalised linear mixed models by simulation. Methods in Ecology and Evolution, 7(4), 493–498. 10.1111/2041-210X.12504

[bibr27-01461672241257139] Greitemeyer T. (2009). Stereotypes of singles: Are singles what we think? European Journal of Social Psychology, 39(3), 368–383. 10.1002/ejsp.542

[bibr28-01461672241257139] Havighurst R. J. (1972). Developmental tasks and education (3rd ed.). McKay.

[bibr29-01461672241257139] Heller D. Watson D. Ilies R. (2004). The role of person versus situation in life satisfaction: A critical examination. Psychological Bulletin, 130(4), 574–600. 10.1037/0033-2909.130.4.574 15250814

[bibr30-01461672241257139] Kislev E. (2018). Happiness, post-materialist values, and the unmarried. Journal of Happiness Studies, 19(8), 2243–2265. 10.1007/s10902-017-9921-7

[bibr31-01461672241257139] Kislev E. (2021). Reduced relationship desire is associated with better life satisfaction for singles in Germany: An analysis of pairfam data. Journal of Social and Personal Relationships, 38(7), 2073–2083. https://10.1177/02654075211005024

[bibr32-01461672241257139] Krings F. Bangerter A. Gomez V. Grob A. (2008). Cohort differences in personal goals and life satisfaction in young adulthood: Evidence for historical shifts in developmental tasks. Journal of Adult Development, 15(2), 93–105. 10.1007/s10804-008-9039-6

[bibr33-01461672241257139] Lee J. Kim H. Woo J. Chang S. M. Hong J. P. Lee D. W. . . . Kim B. S. (2020). Impacts of remaining single above the mean marriage age on mental disorders and suicidality: A nationwide study in Korea. Journal of Korean Medical Science, 35(37), 1–14. 10.3346/jkms.2020.35.e319 PMC7505730 32959544

[bibr34-01461672241257139] Lesthaeghe R. (2014). The second demographic transition: A concise overview of its development. Proceedings of the National Academy of Sciences of the United States of America, 111(51), 18112–18115. 10.1073/pnas.1420441111 25453112 PMC4280616

[bibr35-01461672241257139] Lorah J. (2018). Effect size measures for multilevel models: Definition, interpretation, and TIMSS example. Large-Scale Assessments in Education, 6(1), 8. 10.1186/s40536-018-0061-2

[bibr36-01461672241257139] Mehta C. M. Arnett J. J. Palmer C. G. Nelson L. J. (2020). Established adulthood: A new conception of ages 30 to 45. American Psychologist, 75(4), 431–444. 10.1037/amp0000600 32378940

[bibr37-01461672241257139] Mortelmans D. Claessens E. Thielemans G. (2023). Defining and measuring singlehood in family studies. Journal of Family Theory and Review, 15(3), 485–505. 10.1111/jftr.12520

[bibr38-01461672241257139] Nelson L. J. (2021). The theory of emerging adulthood 20 years later: A look at where it has taken us, what we know now, and where we need to go. Emerging Adulthood, 9(3), 179–188. 10.1177/2167696820950884

[bibr39-01461672241257139] Neyer F. J. Mund M. Zimmermann J. Wrzus C. (2014). Personality-relationship transactions revisited. Journal of Personality, 82(6), 539–550. 10.1111/jopy.12063 23927445

[bibr40-01461672241257139] OECD. (2022). SF3.1: Marriage and divorce rates [PDF file]. https://www.oecd.org/els/family/SF_3_1_Marriage_and_divorce_rates.pdf

[bibr41-01461672241257139] Oh J. Chopik W. J. Lucas R. E. (2022). Happiness singled out: Bidirectional associations between singlehood and life satisfaction. Personality and Social Psychology Bulletin, 48(11), 1597–1613. 10.1177/01461672211049049 34612739

[bibr42-01461672241257139] Ozer D. J. Benet-Martínez V. (2006). Personality and the prediction of consequential outcomes. Annual Review of Psychology, 57, 401–421. 10.1146/annurev.psych.57.102904.190127 16318601

[bibr43-01461672241257139] Park Y. Impett E. A. MacDonald G. (2020). Singles’ sexual satisfaction is associated with more satisfaction with singlehood and less interest in marriage. Personality and Social Psychology Bulletin, 47(5), 741–752. 10.1177/0146167220942361 32779516

[bibr44-01461672241257139] Park Y. Page-Gould E. MacDonald G. (2022). Satisfying singlehood as a function of age and cohort: Satisfaction with being single increases with age after midlife. Psychology and Aging, 37(5), 626–636. 10.1037/pag0000695 35708941

[bibr45-01461672241257139] Poortman A. R. Liefbroer A. C. (2010). Singles’ relational attitudes in a time of individualization. Social Science Research, 39(6), 938–949. 10.1016/j.ssresearch.2010.03.012

[bibr46-01461672241257139] Rammstedt B. John O. P. (2005). Kurzversion des Big Five Inventory (BFI-K): Entwicklung und Validierung eines ökonomischen Inventars zur Erfassung der fünf Faktoren der Persönlichkeit [Short version of the Big Five Inventory (BFI-K): Development and validation of an economical inventory]. Diagnostica, 51(4), 195–206. 10.1026/0012-1924.51.4.195

[bibr47-01461672241257139] R Core Team. (2020). R: A language and environment for statistical computing. R Foundation for Statistical Computing. https://www.r-project.org/

[bibr48-01461672241257139] Reynolds J. Wetherell M. (2003). The discursive climate of singleness: The consequences for women’s negotiation of a single identity. Feminism and Psychology, 13(4), 489–510. 10.1177/09593535030134014

[bibr49-01461672241257139] Schaie K. W. (1965). A general model for the study of developmental problems. Psychological Bulletin, 64(2), 92–107. 10.1037/h0022371 14320080

[bibr50-01461672241257139] Scheling L. Richter D. (2021). Generation Y: Do millennials need a partner to be happy? Journal of Adolescence, 90(1), 23–31. 10.1016/j.adolescence.2021.05.006 34090110

[bibr51-01461672241257139] Seifert I. S. Rohrer J. M. Egloff B. Schmuckle S. C. (2022). The development of the rank-order stability of the Big Five across the life span. Journal of Personality and Social Psychology, 122(5), 920–941. https://10.1037/pspp0000398 35404643 10.1037/pspp0000398

[bibr52-01461672241257139] Seiffge-Krenke I. (2003). Testing theories of romantic development from adolescence to young adulthood: Evidence of a developmental sequence. International Journal of Behavioral Development, 27(6), 519–531. 10.1080/01650250344000145

[bibr53-01461672241257139] Sharp E. A. Ganong L. (2011). “I’m a loser, I’m not married, let’s just all look at me”: Ever-single women’s perceptions of their social environment. Journal of Family Issues, 32(7), 956–980. 10.1177/0192513X10392537

[bibr54-01461672241257139] Smetana J. G. Campione-Barr N. Metzger A. (2006). Adolescent development in interpersonal and societal contexts. Annual Review of Psychology, 57(1), 255–284. 10.1146/annurev.psych.57.102904.190124 16318596

[bibr55-01461672241257139] Spielmann S. S. MacDonald G. Maxwell J. A. Joel S. Peragine D. Muise A. Impett E. A. (2013). Settling for less out of fear of being single. Journal of Personality and Social Psychology, 105(6), 1049–1073. 10.1037/a0034628 24128187

[bibr56-01461672241257139] Stahnke B. Cooley M. (2021). A systematic review of the association between partnership and life satisfaction. Family Journal, 29(2), 182–189. 10.1177/1066480720977517

[bibr57-01461672241257139] Steinberg L. Morris A. S. (2001). Adolescent development. Annual Review of Psychology, 52, 83–110. 10.1146/annurev.psych.52.1.83 11148300

[bibr58-01461672241257139] Tillman K. H. Brewster K. L. Holway G. V. (2019). Sexual and romantic relationships in young adulthood. Annual Review of Sociology, 45, 133–153. 10.1146/annurev-soc-073018-022625

[bibr59-01461672241257139] van de Bongardt D. Reitz E. Sandfort T. Deković M. (2015). A meta-analysis of the relations between three types of peer norms and adolescent sexual behavior. Personality and Social Psychology Review, 19(3), 203–234. 10.1177/1088868314544223 25217363 PMC5871927

[bibr60-01461672241257139] Van Tilburg T. G. Aartsen M. J. van der Pas S . (2015). Loneliness after divorce: A cohort comparison among Dutch young-old adults. European Sociological Review, 31(3), 243–252. 10.1093/esr/jcu086

[bibr61-01461672241257139] Vaterlaus J. M. Tulane S. Porter B. D. Beckert T. E. (2018). The perceived influence of media and technology on adolescent romantic relationships. Journal of Adolescent Research, 33(6), 651–671. 10.1177/0743558417712611

[bibr62-01461672241257139] Weidmann R. Ledermann T. Grob A. (2016). The interdependence of personality and satisfaction in couples: A review. European Psychologist, 21(4), 284–295. 10.1027/1016-9040/a000261

