# Supplementary material for: Today’s Adolescents Are More Satisfied With Being Single: Findings From a German Cohort-Sequential Study Among 14- to 40-Year-Olds
Source: Pers Soc Psychol Bull. 2024 Jun 10;51(12):2461–75. doi: 10.1177/01461672241257139 (PMC12569122; doi:10.1177/01461672241257139)
Supplement: sj-docx-1-psp-10.1177_01461672241257139 – Supplemental material for Today’s Adolescents Are More Satisfied With Being Single: Findings From a German Cohort-Sequential Study Among 14- to 40-Year-Olds [file sj-docx-1-psp-10.1177_01461672241257139.docx]

# Supplementary Material

# The Supplementary Material provides additional information and analyses for the manuscript “Today’s adolescents are more satisfied with being single: Findings from a cohort-sequential study”.

Deviations from the preregistration are detailed on pages 2-3, while descriptive statistics on satisfaction per study wave are presented on page 4. Results from the multilevel analyses can be found on pages 5-11.

We also conducted two additional analyses regarding the cohort effects: descriptive information and results are on pages 12-13 (potential mechanisms behind the cohort effect) and pages 14-16 (historical changes in partnered individuals).

In our preregistration, we aimed to control for single duration, but encountered several issues with this variable (see Table S1). Pages 17-27 cover information regarding the measure of single duration and results from multilevel analyses including this variable.

# Deviations From Preregistration

Our final analyses included a number of deviations from the preregistered plan (https://osf.io/8xf95/?view_only=44d42dd0ef33490299666b63c3deeac2). These deviations are listed in Table S1.

**Table S1**

*Deviations of the Current Study From the Preregistration*

| Registered plan | Deviation | Reason for deviation |
| --- | --- | --- |
| “To further explore the role of the Big Five personality traits, data from Waves 2, 6, 10, and 11 will be used as these Waves included measures on participants’ personality traits.” | We did not use data from Wave 6. | Data from Wave 6 were not necessary because we obtained information on participants’ Big Five personality traits from Wave 2 for those who participated in Waves 1-3, from Wave 10 for those who participated in Waves 11-13, and from Wave 11 for participants from the refreshment sample. |
| “To test the robustness of the results, we will rerun all models and include the covariates participants’ duration of being single (all models) and sex (except in Model 4) as additional predictors at level 2. In particular, we will compare direction and statistical significance of coefficients in the model without covariates and in the model with covariates. Only coefficients that are significant and show effects in the same direction in both models will be interpreted as robust effects.” | We included only participants’ gender as an additional covariate. We interpreted coefficients that were significant and showed effects in the same direction in both models (with and without covariate). | There were several issues with the variable duration of being single. As the duration of being single describes the time (in months) that has passed since the end of the last romantic relationship and most participants were not in a romantic relationship before T1 (*n* = 1,892, 61.9%), we had information on single duration only for *N* = 1,130 (35.7%) participants. Ultimately, results of these control analyses were underpowered and only generalizable to people with past romantic relationship experience. More information regarding the measure of single duration and results including both covariates are reported in the Supplementary Material (pages 17-27). |
| “We will be using full information maximum likelihood procedures to deal with incomplete data.” | We used listwise deletion instead. | The *Lmer* function cannot deal with missing data. When fitting LME models with R packages, listwise deletion (i.e., deletion of rows with missing response) is used. Overall, the proportion of missing data was negligible ranging from 0.03% (life satisfaction at T1) to 1.77% (singlehood satisfaction at T1). |
| “Model 2: Prevalence effects (H3). The second model tests whether the prevalence of singles at T1 is related to singles’ well-being. This will be done by extending Model 1 by the prevalence of singles as an additional predictor at level 2 as well as an interaction term with time (cross- level).” | We computed Spearman’s rank correlations to assess the association between the proportion of singles and satisfaction. | Estimating the effect of proportion on singles’ satisfaction with a multilevel growth-curve model resulted in estimation problems due to high multicollinearity between the predictors (VIFs ranging from 5.9 – 74.0). In addition, as we were particularly interested in the cross- sectional association between prevalence of singles and satisfaction at T1, Spearman’s rank correlation was the most straightforward and efficient approach to test this hypothesis. |

# Descriptive Statistics on Satisfaction per Study Wave

**Table S2**

*Means and Standard Deviations of Singlehood Satisfaction and Life Satisfaction Separated by Cohort and Age Group*

| Birth cohort | Age group | T1 Life satisfaction *M* (*SD*) | T2 Life satisfaction *M* (*SD*) | T3 Life satisfaction *M* (*SD*) | T1 Singlehood satisfaction *M* (*SD*) | T2 Singlehood satisfaction *M* (*SD*) | T3 Singlehood satisfaction *M* (*SD*) |
| --- | --- | --- | --- | --- | --- | --- | --- |
| Earlier-born | Adolescents | 7.94 (1.49) | 7.96 (1.54) | 7.74 (1.53) | 6.93 (2.26) | 6.75 (2.42) | 6.63 (2.36) |
|  | Emerging adults | 6.80 (1.90) | 6.89 (1.94) | 6.66 (1.88) | 6.14 (2.32) | 5.54 (2.52) | 5.67 (2.41) |
|  | Established adults | 6.51 (2.17) | 6.63 (1.99) | 6.55 (2.00) | 6.12 (2.47) | 5.99 (2.56) | 5.98 (2.63) |
| Later-born | Adolescents | 8.04 (1.52) | 7.67 (1.48) | 7.40 (1.60) | 7.46 (2.23) | 7.16 (2.21) | 7.05 (2.08) |
|  | Emerging adults | 6.94 (1.49) | 7.05 (1.65) | 6.81 (1.63) | 6.16 (2.43) | 5.95 (2.40) | 6.09 (2.34) |
|  | Established adults | 6.65 (1.81) | 6.58 (1.80) | 6.75 (1.87) | 5.73 (2.72) | 5.52 (2.70) | 5.80 (2.73) |

*Note*. The earlier-born cohorts included adolescents born in 1991-1993, emerging adults born in 1981-1983, and established adults born in 1971-1973. Assessment periods for this cohort were T1 in 2008/2009, T2 in 2009/2019, and T3 in 2010/2011. The later-born cohorts included adolescents born in 2001-2003, emerging adults born in 1991-1993, and established adults born in 1981-1983. Assessment periods for this cohort were T1 in 2018/2019, T2 in 2019/2020, and T3 in 2020/2021.

**Table S3**

*Model 1: Intraindividual Change and Age Effects in Singlehood Satisfaction and Life Satisfaction*

|  | Singlehood satisfaction | | | | | | Life satisfaction | | | | | | |
| --- | --- | --- | --- | --- | --- | --- | --- | --- | --- | --- | --- | --- | --- |
|  | Without control variable | | | With control variable | | | Without control variable | | | With control variable | | | |
|  | *b* | *SE* | *p* | *b* | *SE* | *p* | *b* | *SE* | *p* | *b* | *SE* | *p* |  |
| Intercept | **7.12** | 0.05 | <.001 | **6.96** | 0.06 | <.001 | **8.00** | 0.04 | <.001 | **8.07** | 0.04 | <.001 |  |
| Time | **-0.17** | 0.03 | <.001 | **-0.17** | 0.03 | <.001 | **-0.19** | 0.02 | <.001 | **-0.19** | 0.02 | <.001 |  |
| Emerging adulthood | **-1.07** | 0.11 | <.001 | **-1.07** | 0.11 | <.001 | **-1.10** | 0.08 | <.001 | **-1.10** | 0.08 | <.001 |  |
| Established adulthood | **-1.21** | 0.12 | <.001 | **-1.25** | 0.12 | <.001 | **-1.42** | 0.08 | <.001 | **-1.40** | 0.08 | <.001 |  |
| Time x emerging adulthood | 0.00 | 0.06 | .987 | 0.00 | 0.06 | .987 | **0.12** | 0.04 | .006 | **0.12** | 0.04 | .006 |  |
| Time x established adulthood | 0.15 | 0.07 | .030 | 0.15 | 0.07 | .030 | **0.23** | 0.05 | <.001 | **0.23** | 0.05 | <.001 |  |
| Gender |  |  |  | **0.44** | 0.07 | <.001 |  |  |  | **-0.16** | 0.05 | .001 |  |

*Note*. Level 1 sample sizes ranged from *N* = 8,667 for singlehood satisfaction to *N* = 8,801 for life satisfaction. Significant parameters are displayed in bold (*p* ≤ .01).

**Table S4**

*Model 2: Cohort Effects on Singlehood Satisfaction and Life Satisfaction*

|  | Singlehood satisfaction | | | | | | Life satisfaction | | | | | | |
| --- | --- | --- | --- | --- | --- | --- | --- | --- | --- | --- | --- | --- | --- |
|  | Without control variable | | | With control variable | | | | Without control variable | | | With control variable | | |
|  | *b* | *SE* | *p* | *b* | *SE* | *p* | | *b* | *SE* | *p* | *b* | *SE* | *p* |
| Intercept | **6.95** | 0.07 | <.001 | **6.79** | 0.07 | <.001 | | **8.01** | 0.05 | <.001 | **8.08** | 0.05 | <.001 |
| Time | **-0.18** | 0.04 | <.001 | **-0.18** | 0.04 | <.001 | | **-0.13** | 0.03 | <.001 | **-0.13** | 0.03 | <.001 |
| Emerging adulthood | **-0.99** | 0.13 | <.001 | **-0.98** | 0.13 | <.001 | | **-1.21** | 0.09 | <.001 | **-1.22** | 0.09 | <.001 |
| Established adulthood | **-0.89** | 0.14 | <.001 | **-0.94** | 0.14 | <.001 | | **-1.54** | 0.10 | <.001 | **-1.53** | 0.10 | <.001 |
| Later-born cohort | **0.43** | 0.10 | <.001 | **0.39** | 0.10 | <.001 | | -0.04 | 0.07 | .620 | -0.03 | 0.07 | .684 |
| Time x emerging adulthood | 0.00 | 0.06 | .994 | 0.00 | 0.06 | .993 | | 0.11 | 0.04 | .011 | 0.11 | 0.04 | .011 |
| Time x established adulthood | 0.15 | 0.07 | .028 | 0.15 | 0.07 | .028 | | **0.22** | 0.05 | <.001 | **0.22** | 0.05 | <.001 |
| Time x later-born cohort | 0.02 | 0.05 | .710 | 0.02 | 0.05 | .708 | | **-0.14** | 0.03 | <.001 | **-0.14** | 0.03 | <.001 |
| Emerging adulthood x later-born cohort | -0.17 | 0.19 | .370 | -0.18 | 0.19 | .330 | | 0.33 | 0.13 | .015 | 0.34 | 0.13 | .011 |
| Established adulthood x later-born cohort | **-0.80** | 0.20 | .001 | **-0.77** | 0.20 | <.001 | | 0.31 | 0.14 | .029 | 0.30 | 0.14 | .032 |
| Gender |  |  |  | **0.42** | 0.07 | <.001 | |  |  |  | **-0.16** | 0.05 | .002 |

*Note*. Level 1 sample sizes ranged from *N* = 8,667 for singlehood satisfaction to *N* = 8,801 for life satisfaction. Significant parameters are displayed in bold (*p* ≤ .01).

**Table S5**

*Model 3: Gender Effects on Singlehood Satisfaction and Life Satisfaction*

|  | Singlehood satisfaction | | | Life satisfaction | | |
| --- | --- | --- | --- | --- | --- | --- |
|  | *b* | *SE* | *p* | *b* | *SE* | *p* |
| Intercept | **6.89** | 0.08 | <.001 | **8.09** | 0.06 | <.001 |
| Time | **-0.16** | 0.04 | <.001 | **-0.13** | 0.03 | <.001 |
| Emerging adulthood | **-1.06** | 0.14 | <.001 | **-1.28** | 0.10 | <.001 |
| Established adulthood | **-1.14** | 0.17 | <.001 | **-1.68** | 0.12 | <.001 |
| Later-born cohort | 0.22 | 0.12 | .055 | 0.02 | 0.08 | .768 |
| Gender | 0.18 | 0.12 | .125 | -0.18 | 0.08 | .027 |
| Time x emerging adulthood | 0.00 | 0.06 | .999 | 0.11 | 0.04 | .011 |
| Time x established adulthood | 0.15 | 0.07 | .025 | **0.23** | 0.05 | <.001 |
| Time x later-born cohort | 0.02 | 0.05 | .672 | **-0.14** | 0.03 | <.001 |
| Time x gender | -0.04 | 0.05 | .392 | -0.01 | 0.03 | .756 |
| Emerging adulthood x later-born cohort | -0.20 | 0.19 | .292 | 0.32 | 0.13 | .016 |
| Established adulthood x later-born cohort | **-0.80** | 0.20 | <.001 | 0.31 | 0.14 | .030 |
| Emerging adulthood x gender | 0.19 | 0.19 | .314 | 0.17 | 0.13 | .184 |
| Established adulthood x gender | 0.47 | 0.20 | .016 | 0.32 | 0.14 | .023 |
| Later-born cohort x gender | **0.42** | 0.14 | .003 | -0.12 | 0.10 | .257 |

*Note*. Level 1 sample sizes ranged from *N* = 8,664 for singlehood satisfaction to *N* = 8,798 for life satisfaction. Significant parameters are displayed in bold (*p* ≤ .01).

**Table S6**

*Model 4: Extraversion Effects on Singlehood Satisfaction and Life Satisfaction*

|  | Singlehood satisfaction | | | | | | Life satisfaction | | | | | | |
| --- | --- | --- | --- | --- | --- | --- | --- | --- | --- | --- | --- | --- | --- |
|  | Without control variable | | | With control variable | | | | Without control variable | | | With control variable | | |
|  | *b* | *SE* | *p* | *b* | *SE* | *p* | | *b* | *SE* | *p* | *b* | *SE* | *p* |
| Intercept | **6.15** | 0.21 | <.001 | **6.13** | 0.21 | <.001 | | **6.56** | 0.15 | <.001 | **6.62** | 0.15 | <.001 |
| Time | -0.24 | 0.10 | .014 | -0.25 | 0.10 | .013 | | 0.02 | 0.07 | .729 | 0.03 | 0.07 | .693 |
| Emerging adulthood | **-0.95** | 0.13 | <.001 | **-0.95** | 0.13 | <.001 | | **-1.15** | 0.09 | <.001 | **-1.16** | 0.09 | <.001 |
| Established adulthood | **-0.85** | 0.14 | <.001 | **-0.91** | 0.14 | <.001 | | **-1.50** | 0.10 | <.001 | **-1.48** | 0.10 | <.001 |
| Later-born cohort | **1.01** | 0.28 | <.001 | **0.96** | 0.28 | .001 | | **0.63** | 0.20 | .001 | **0.62** | 0.20 | .002 |
| Extraversion | **0.23** | 0.06 | <.001 | **0.20** | 0.06 | .001 | | **0.43** | 0.04 | <.001 | **0.44** | 0.04 | <.001 |
| Time x emerging adulthood | 0.00 | 0.06 | .993 | 0.00 | 0.06 | .996 | | 0.11 | 0.04 | .014 | 0.11 | 0.04 | .014 |
| Time x established adulthood | 0.14 | 0.07 | .032 | 0.14 | 0.07 | .032 | | **0.22** | 0.05 | <.001 | **0.22** | 0.05 | <.001 |
| Time x later-born cohort | 0.02 | 0.05 | .714 | 0.02 | 0.05 | .711 | | **-0.15** | 0.03 | <.001 | **-0.15** | 0.03 | <.001 |
| Time x extraversion | 0.02 | 0.03 | .467 | 0.02 | 0.03 | .448 | | -0.05 | 0.02 | .016 | -0.05 | 0.02 | .014 |
| Emerging adulthood x later-born cohort | -0.24 | 0.19 | .215 | -0.24 | 0.19 | .208 | | 0.21 | 0.13 | .112 | 0.22 | 0.13 | .094 |
| Established adulthood x later-born cohort | **-0.84** | 0.20 | <.001 | **-0.80** | 0.20 | <.001 | | 0.25 | 0.14 | .070 | 0.24 | 0.14 | .089 |
| Later-born cohort x extraversion | -0.17 | 0.08 | .038 | -0.16 | 0.08 | .040 | | **-0.18** | 0.06 | .001 | **-0.17** | 0.06 | .002 |
| Gender |  |  |  | **0.39** | 0.07 | <.001 | |  |  |  | **-0.23** | 0.05 | <.001 |

*Note*. Level 1 sample sizes ranged from *N* = 8,640 for singlehood satisfaction to *N* = 8,774 for life satisfaction. Significant parameters are displayed in bold (*p* ≤ .01).

**Table S7**

*Model 4: Neuroticism Effects on Singlehood Satisfaction and Life Satisfaction*

|  | Singlehood satisfaction | | | | | | Life satisfaction | | | | | | |
| --- | --- | --- | --- | --- | --- | --- | --- | --- | --- | --- | --- | --- | --- |
|  | Without control variable | | | With control variable | | | | Without control variable | | | With control variable | | |
|  | *b* | *SE* | *p* | *b* | *SE* | *p* | | *b* | *SE* | *p* | *b* | *SE* | *p* |
| Intercept | **8.41** | 0.17 | <.001 | **8.33** | 0.17 | <.001 | | **9.85** | 0.12 | <.001 | **9.84** | 0.12 | <.001 |
| Time | -0.15 | 0.08 | .072 | -0.15 | 0.08 | .074 | | **-0.24** | 0.06 | <.001 | **-0.24** | 0.06 | <.001 |
| Emerging adulthood | **-0.95** | 0.13 | <.001 | **-0.93** | 0.12 | <.001 | | **-1.16** | 0.09 | <.001 | **-1.16** | 0.09 | <.001 |
| Established adulthood | **-0.79** | 0.14 | <.001 | **-0.85** | 0.14 | <.001 | | **-1.43** | 0.10 | <.001 | **-1.44** | 0.10 | <.001 |
| Later-born cohort | -0.13 | 0.25 | .615 | -0.06 | 0.25 | .812 | | -0.17 | 0.17 | .303 | -0.16 | 0.17 | .330 |
| Neuroticism | **-0.56** | 0.06 | <.001 | **-0.61** | 0.06 | <.001 | | **-0.70** | 0.04 | <.001 | **-0.70** | 0.04 | <.001 |
| Time x emerging adulthood | 0.00 | 0.06 | .983 | 0.00 | 0.06 | .984 | | 0.11 | 0.04 | .012 | 0.11 | 0.04 | .013 |
| Time x established adulthood | 0.14 | 0.07 | .033 | 0.14 | 0.07 | .034 | | **0.22** | 0.05 | <.001 | **0.22** | 0.05 | <.001 |
| Time x later-born cohort | 0.02 | 0.05 | .707 | 0.02 | 0.05 | .707 | | **-0.15** | 0.04 | <.001 | **-0.15** | 0.04 | <.001 |
| Time x neuroticism | -0.01 | 0.03 | .750 | -0.01 | 0.03 | .742 | | 0.04 | 0.02 | .042 | 0.04 | 0.02 | .041 |
| Emerging adulthood x later-born cohort | -0.22 | 0.19 | .229 | -0.25 | 0.18 | .183 | | 0.28 | 0.12 | .027 | 0.28 | 0.12 | .025 |
| Established adulthood x later-born cohort | **-0.88** | 0.20 | <.001 | **-0.82** | 0.20 | <.001 | | 0.22 | 0.13 | .090 | 0.23 | 0.13 | .077 |
| Later-born cohort x neuroticism | **0.24** | 0.08 | .005 | 0.21 | 0.08 | .014 | | 0.11 | 0.06 | .056 | 0.10 | 0.06 | .070 |
| Gender |  |  |  | **0.60** | 0.07 | <.001 | |  |  |  | 0.05 | 0.05 | .300 |

*Note*. Level 1 sample sizes ranged from *N* = 8,643 for singlehood satisfaction to *N* = 8,777 for life satisfaction. Significant parameters are displayed in bold (*p* ≤ .01).

| **Table S8** |  | | | | | |  | | | | | | | |
| --- | --- | --- | --- | --- | --- | --- | --- | --- | --- | --- | --- | --- | --- | --- |
| *Model 4: Effects of Big Five Personality Traits on Singlehood Satisfaction and Life Satisfaction* | | | | | | | | | | | | | | |
|  | Singlehood satisfaction | | | | | | Life satisfaction | | | | | | | |
|  | Without control variable | | | With control variable | | | | Without control variable | | | With control variable | | | |
|  | *b* | *SE* | *p* | *b* | *SE* | *p* | | *b* | *SE* | *p* | *b* | *SE* | *p* |  |
| Intercept | **8.00** | 0.46 | <.001 | **8.58** | 0.46 | <.001 | | **7.68** | 0.31 | <.001 | **7.61** | 0.31 | <.001 |  |
| Time | -0.46 | 0.22 | .035 | -0.47 | 0.22 | .033 | | -0.06 | 0.16 | .682 | -0.06 | 0.16 | .698 |  |
| Emerging adulthood | **-0.98** | 0.13 | <.001 | **-0.96** | 0.13 | <.001 | | **-1.22** | 0.09 | <.001 | **-1.23** | 0.09 | <.001 |  |
| Established adulthood | **-0.87** | 0.14 | <.001 | **-0.91** | 0.14 | <.001 | | **-1.56** | 0.10 | <.001 | **-1.56** | 0.10 | <.001 |  |
| Later-born cohort | -0.13 | 0.63 | .843 | 0.00 | 0.63 | .999 | | -0.08 | 0.41 | .845 | -0.12 | 0.41 | .768 |  |
| Extraversion | 0.10 | 0.06 | .097 | 0.05 | 0.06 | .401 | | **0.23** | 0.04 | <.001 | **0.23** | 0.04 | <.001 |  |
| Neuroticism | **-0.49** | 0.06 | <.001 | **-0.58** | 0.07 | <.001 | | **-0.57** | 0.04 | <.001 | **-0.56** | 0.04 | <.001 |  |
| Agreeableness | -0.03 | 0.07 | .666 | -0.05 | 0.07 | .425 | | 0.09 | 0.04 | .053 | 0.09 | 0.04 | .041 |  |
| Conscientiousness | **0.19** | 0.07 | .007 | 0.14 | 0.07 | .046 | | **0.31** | 0.05 | <.001 | **0.31** | 0.05 | <.001 |  |
| Openness | **-0.19** | 0.07 | .009 | **-0.23** | 0.07 | .001 | | -0.08 | 0.05 | .104 | -0.07 | 0.05 | .149 |  |
| Time x emerging adulthood | -0.02 | 0.06 | .742 | -0.02 | 0.06 | .745 | | **0.12** | 0.04 | .009 | **0.12** | 0.04 | .009 |  |
| Time x established adulthood | 0.12 | 0.07 | .081 | 0.12 | 0.07 | .081 | | **0.23** | 0.05 | <.001 | **0.23** | 0.05 | <.001 |  |
| Time x later-born cohort | 0.02 | 0.05 | .678 | 0.02 | 0.05 | .675 | | **-0.15** | 0.04 | <.001 | **-0.15** | 0.04 | <.001 |  |
| Time x extraversion | 0.00 | 0.03 | .958 | 0.00 | 0.03 | .933 | | -0.04 | 0.02 | .090 | -0.04 | 0.02 | .084 |  |
| Time x neuroticism | 0.00 | 0.03 | .878 | 0.00 | 0.03 | .877 | | 0.03 | 0.02 | .172 | 0.03 | 0.02 | .170 |  |
| Time x agreeableness | -0.02 | 0.03 | .557 | -0.02 | 0.03 | .563 | | 0.03 | 0.02 | .250 | 0.03 | 0.02 | .248 |  |
| Time x conscientiousness | 0.06 | 0.03 | .085 | 0.06 | 0.03 | .085 | | -0.03 | 0.02 | .223 | -0.03 | 0.02 | .216 |  |
| Time x openness | 0.04 | 0.03 | .258 | 0.04 | 0.03 | .257 | | 0.00 | 0.02 | .939 | 0.00 | 0.02 | .947 |  |
| Emerging adulthood x later-born cohort | -0.19 | 0.19 | .303 | -0.22 | 0.19 | .241 | | **0.32** | 0.12 | .010 | **0.32** | 0.12 | .008 |  |
| Established adulthood x later-born cohort | **-0.83** | 0.20 | <.001 | **-0.78** | 0.20 | <.001 | | 0.26 | 0.13 | .043 | 0.26 | 0.13 | .045 |  |
| Later-born cohort x extraversion | -0.12 | 0.09 | .146 | -0.13 | 0.08 | .122 | | **-0.15** | 0.06 | .005 | **-0.15** | 0.06 | .008 |  |
| Later-born cohort x neuroticism | 0.20 | 0.09 | .025 | 0.17 | 0.09 | .063 | | 0.07 | 0.06 | .254 | 0.07 | 0.06 | .225 |  |
| Later-born cohort x agreeableness | 0.04 | 0.09 | .647 | 0.02 | 0.09 | .841 | | **0.17** | 0.06 | .005 | **0.17** | 0.06 | .004 |  |
| Later-born cohort x conscientiousness | -0.06 | 0.10 | .581 | -0.07 | 0.10 | .489 | | -0.01 | 0.07 | .890 | 0.00 | 0.07 | .962 |  |
| Later-born cohort x openness | 0.16 | 0.10 | .111 | 0.18 | 0.10 | .067 | | 0.02 | 0.07 | .792 | 0.01 | 0.07 | .865 |  |
| Gender |  |  |  | **0.60** | 0.07 | <.001 | |  |  |  | -0.09 | 0.05 | .062 |  |

*Note*. Level 1 sample sizes ranged from *N* = 8,634 for singlehood satisfaction to *N* = 8,768 for life satisfaction. Significant parameters are displayed in bold (*p* ≤ .01).

# Potential Mechanisms Behind the Cohort Effect

Based on valuable feedback we received during the review process, we explored three potential mechanisms that could have contributed to increased satisfaction with singlehood among adolescents nowadays: *desire for a romantic relationship* (“I would like to have a partner”, ranging from 1 [not at all] to 5 [absolutely]), *sexual satisfaction* (“How satisfied are you with your sex life?”, ranging from 0 [very dissatisfied] to 10 [very satisfied]), and *friendship satisfaction* (“How satisfied are you with the following domains in your life: friends, social contacts?”, ranging from 0 [very dissatisfied] to 10 [very satisfied]) at T1. Table S9 shows the means, standard deviations, and zero-order correlations among these factors and satisfaction at T1.

**Table S9**

*Descriptive Characteristics and Pearson Correlations Among the Study Variables*

|  | 1 | 2 | 3 | 4 | 5 |
| --- | --- | --- | --- | --- | --- |
| 1. Desire for a romantic relationship (*n* = 2,851) |  |  |  |  |  |
| 2. Sexual satisfaction (*n* = 2,112) | -.30 |  |  |  |  |
| 3. Friendship satisfaction (*n* = 2,935) | -.11 | .24 |  |  |  |
| 4. Singlehood satisfaction (*n* = 2,883) | -.54 | .36 | .22 |  |  |
| 5. Life satisfaction (*n* = 2,935) | -.16 | .27 | .39 | .34 |  |
| *M* | 3.37 | 4.95 | 8.18 | 6.77 | 7.55 |
| *SD* | 1.20 | 2.77 | 1.92 | 2.39 | 1.78 |

*Note*. All correlations were significant at *p* < .001.

We conducted *t*-tests to test cohort differences in these factors and calculated three additional multilevel models to assess both the main effects of these factors and their interaction effects with cohort membership on singlehood satisfaction.

Among these factors, the desire for a romantic relationship stood out as a probable contributor to the historical increase in singlehood satisfaction among adolescents. Specifically, adolescents born in 2001-2003 reported a lower desire for a romantic relationship (*M* = 3.01, *SD* = 1.13) compared to those born in 1991-1993 (*M* = 3.38, *SD* = 1.17), *t*(1654) = -6.78, *p* < .001, *d* = -0.32. Furthermore, adolescents with a higher desire for a romantic relationship were less satisfied with being single (*b* = -0.92, 95% CI [-0.99, -0.85], *p* < .001), suggesting that the overall decline in romantic desire may have benefitted adolescents’ satisfaction.

Neither sexual satisfaction nor friendship satisfaction could explain the trend towards increased singlehood satisfaction. While singles who reported higher levels of sexual satisfaction also reported greater satisfaction with being single (*b* = 0.26, 95% CI [0.22, 0.30], *p* < .001), there was no significant difference in sexual satisfaction between adolescents born in 2001-2003 (*M* = 5.62, *SD* = 2.73) and those born in 1991-1993 (*M* = 5.36, *SD* = 2.72), *t*(1068) = 1.64, *p* = .101.

Regarding friendship satisfaction, similarly, singles who were more satisfied with friendships and social contacts were also more satisfied with being single (*b* = 0.21, 95% CI [0.16, 0.26], *p* < .001). However, adolescents, on average, experienced a decline in friendship satisfaction over time. Specifically, adolescents born in 2001-2003 reported lower friendship satisfaction (*M* = 8.44, *SD* = 1.61) compared to those born in 1991-1993 (*M* = 8.62, *SD* = 1.63), *t*(1711) = -2.31, *p* = .021, although the effect size was negligible (*d* = 0.11).

# Descriptives and Historical Change Among Partnered Individuals

**Table S10**

*Descriptive Information of the Sample of Partnered Individuals Separated by Cohort and Age Group*

|  | Overall | Adolescents | Emerging adults | Established adults |
| --- | --- | --- | --- | --- |
| *Earlier-born cohort* |  |  |  |  |
| Sample size | 3,118 | 200 | 1,149 | 1,769 |
| Percentage female | 59 | 68 | 60 | 57 |
| *M* age (*SD*) | 31.20 (6.19) | 16.37 (0.83) | 26.16 (0.88) | 36.19 (0.88) |
| *M* relationship satisfaction (*SD*) | 8.29 (2.16) | 8.88 (1.87) | 8.27 (2.28) | 8.24 (2.10) |
| *M* life satisfaction (*SD*) | 7.80 (1.58) | 8.15 (1.41) | 7.75 (1.59) | 7.80 (1.58) |
| *M* extraversion (*SD*) | 3.59 (0.81) | 3.79 (0.84) | 3.63 (0.80) | 3.55 (0.82) |
| *M* neuroticism (*SD*) | 2.67 (0.80) | 2.74 (0.84) | 2.72 (0.82) | 2.63 (0.78) |
| *Later-born cohort* |  |  |  |  |
| Sample size | 2,063 | 140 | 876 | 1,047 |
| Percentage female | 58 | 64 | 59 | 57 |
| *M* age (*SD*) | 30.60 (6.16) | 16.75 (0.85) | 26.14 (0.92) | 36.14 (0.93) |
| *M* relationship satisfaction (*SD*) | 8.22 (1.91) | 9.10 (1.10) | 8.46 (1.61) | 7.89 (2.14) |
| *M* life satisfaction (*SD*) | 7.94 (1.39) | 8.30 (1.26) | 7.95 (1.40) | 7.89 (1.39) |
| *M* extraversion (*SD*) | 3.50 (0.86) | 3.65 (0.90) | 3.50 (0.86) | 3.48 (0.85) |
| *M* neuroticism (*SD*) | 2.79 (0.80) | 2.95 (0.80) | 2.81 (0.83) | 2.75 (0.77) |

*Note*. Age, relationship satisfaction, and life satisfaction refer to T1 (Wave 1 for earlier-born cohort and Wave 11 for later-born cohort). Extraversion and neuroticism were assessed in Waves 2 (for earlier-born cohort), 10 (for later-born emerging and established adults), and 11 (for later-born adolescents). Relationship satisfaction and life satisfaction ranged from 0 to 10. Extraversion and neuroticism ranged from 1 to 5.

**Table S11**

*Model 2: Cohort Effects on Relationship Satisfaction and Life Satisfaction*

|  | Relationship satisfaction | | | | | | Life satisfaction | | | | | | |
| --- | --- | --- | --- | --- | --- | --- | --- | --- | --- | --- | --- | --- | --- |
|  | Without control variable | | | With control variable | | | | Without control variable | | | With control variable | | |
|  | *b* | *SE* | *p* | *b* | *SE* | *p* | | *b* | *SE* | *p* | *b* | *SE* | *p* |
| Intercept | **8.98** | 0.13 | <.001 | **9.05** | 0.13 | <.001 | | **8.25** | 0.10 | <.001 | **8.24** | 0.10 | <.001 |
| Time | **-0.43** | 0.07 | <.001 | **-0.43** | 0.07 | <.001 | | **-0.13** | 0.04 | .003 | **-0.13** | 0.04 | .003 |
| Emerging adulthood | **-0.67** | 0.14 | <.001 | **-0.68** | 0.14 | <.001 | | **-0.47** | 0.10 | <.001 | **-0.47** | 0.10 | <.001 |
| Established adulthood | **-0.81** | 0.13 | <.001 | **-0.82** | 0.13 | <.001 | | **-0.45** | 0.10 | <.001 | **-0.45** | 0.10 | <.001 |
| Later-born cohort | 0.02 | 0.17 | .906 | 0.02 | 0.17 | .920 | | -0.03 | 0.13 | .819 | -0.03 | 0.13 | .823 |
| Time x emerging adulthood | **0.27** | 0.07 | <.001 | **0.27** | 0.07 | <.001 | | **0.13** | 0.05 | .006 | **0.13** | 0.05 | .006 |
| Time x established adulthood | **0.24** | 0.07 | <.001 | **0.24** | 0.07 | <.001 | | 0.08 | 0.05 | .084 | 0.08 | 0.05 | .084 |
| Time x later-born cohort | **0.13** | 0.03 | <.001 | **0.13** | 0.03 | <.001 | | **-0.10** | 0.02 | <.001 | **-0.10** | 0.02 | <.001 |
| Emerging adulthood x later-born cohort | 0.03 | 0.18 | .867 | 0.03 | 0.18 | .862 | | 0.22 | 0.14 | .121 | 0.22 | 0.14 | .121 |
| Established adulthood x later-born cohort | -0.28 | 0.18 | .115 | -0.28 | 0.18 | .119 | | 0.16 | 0.14 | .247 | 0.16 | 0.14 | .249 |
| Gender |  |  |  | -0.10 | 0.04 | .018 | |  |  |  | 0.02 | 0.03 | .534 |

*Note*. Level 1 sample sizes ranged from *N* = 15,154 for relationship satisfaction to *N* = 15,528 for life satisfaction. Significant parameters are displayed in bold (*p* ≤ .01).

**Figure S1**

*Interaction Effect Between Age Group and Cohort Membership on Relationship Satisfaction*


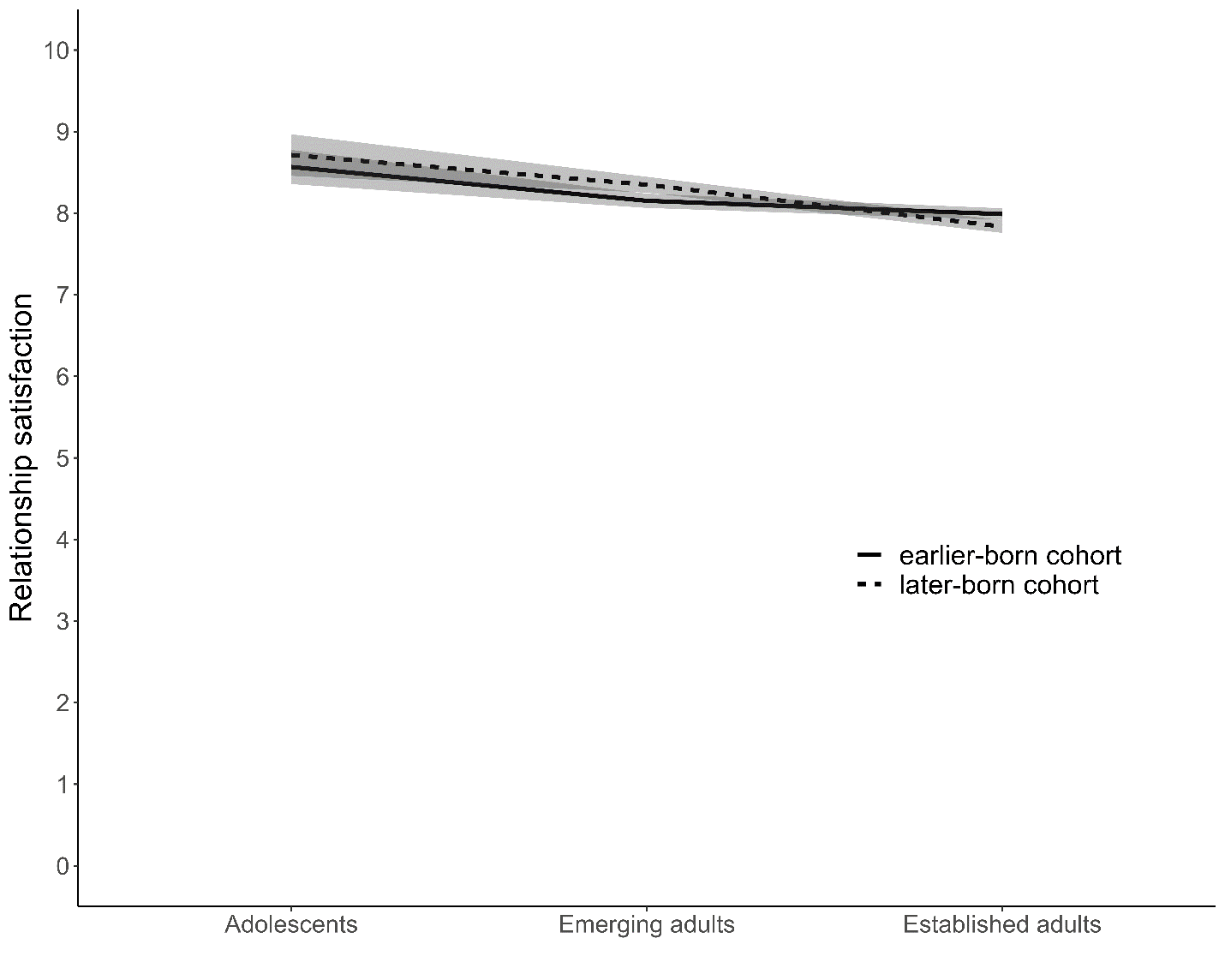


*Note*. Shaded areas represent 95% confidence intervals.

# Single Duration as Additional Control Variable

We reran all models with the covariates participants’ duration of being single (all models) and gender (except in Model 3) as additional predictors at Level 2. The duration of being single was assessed by means of an event-history calendar (EHC; Belli & Callegaro, 2009), a widely used instrument based on a graphical time frame that uses visual cues to facilitate autobiographic memory retrieval. In each study wave, participants were asked to report important life events, including the beginning and end of romantic relationships, on a detailed monthly calendar. The duration of being single described the time that had passed since the end of the last romantic relationship up to T1.

Participants who provided information on their single duration (*N* = 1,130; 35.7%) were on average single about 3.5 years at T1 ranging from less than one month to 262 months (21.8 years) since the last romantic relationship. Results of the analyses with and without the covariates are shown in Tables S12 - S17.

**Table S12**

*Model 1: Intraindividual Change and Age Effects in Singlehood satisfaction and Life Satisfaction*

|  | Singlehood satisfaction | | | | | | Life satisfaction | | | | | | |
| --- | --- | --- | --- | --- | --- | --- | --- | --- | --- | --- | --- | --- | --- |
|  | Without control variables | | | With control variables | | | Without control variables | | | With control variables | | | |
|  | *b* | *SE* | *p* | *b* | *SE* | *p* | *b* | *SE* | *p* | *b* | *SE* | *p* |  |
| Intercept | **7.12** | 0.05 | <.001 | **6.58** | 0.14 | <.001 | **8.00** | 0.04 | <.001 | **8.06** | 0.11 | <.001 |  |
| Time | **-0.17** | 0.03 | <.001 | -0.10 | 0.08 | .169 | **-0.19** | 0.02 | <.001 | **-0.19** | 0.06 | .001 |  |
| Emerging adulthood | **-1.07** | 0.11 | <.001 | **-1.00** | 0.18 | <.001 | **-1.10** | 0.08 | <.001 | **-1.07** | 0.14 | <.001 |  |
| Established adulthood | **-1.21** | 0.12 | <.001 | **-1.15** | 0.20 | <.001 | **-1.42** | 0.08 | <.001 | **-1.51** | 0.15 | <.001 |  |
| Time x emerging adulthood | 0.00 | 0.06 | .987 | 0.05 | 0.10 | .590 | **0.12** | 0.04 | .006 | 0.10 | 0.08 | .200 |  |
| Time x established adulthood | 0.15 | 0.07 | .030 | 0.07 | 0.10 | .467 | **0.23** | 0.05 | <.001 | **0.25** | 0.08 | .001 |  |
| Single duration |  |  |  | 0.00 | 0.00 | .116 |  |  |  | 0.00 | 0.00 | .671 |  |
| Gender |  |  |  | **0.60** | 0.13 | <.001 |  |  |  | -0.07 | 0.09 | .416 |  |

*Note*. For singlehood satisfaction, Level 1 sample sizes ranged from *N* = 2,984 (with control variables) to *N* = 8,667 (without control variables). For life satisfaction, Level 1 sample sizes ranged from *N* = 3,035 to *N* = 8,801 (without control variables). Significant parameters are displayed in bold (*p* ≤ .01).

**Table S13**

*Model 2: Cohort Effects on Singlehood Satisfaction and Life Satisfaction*

|  | Singlehood satisfaction | | | | | | Life satisfaction | | | | | | |
| --- | --- | --- | --- | --- | --- | --- | --- | --- | --- | --- | --- | --- | --- |
|  | Without control variables | | | With control variables | | | | Without control variables | | | With control variables | | |
|  | *b* | *SE* | *p* | *b* | *SE* | *p* | | *b* | *SE* | *p* | *b* | *SE* | *p* |
| Intercept | **6.95** | 0.07 | <.001 | **6.44** | 0.17 | <.001 | | **8.01** | 0.05 | <.001 | **8.12** | 0.13 | <.001 |
| Time | **-0.18** | 0.04 | <.001 | -0.13 | 0.08 | .109 | | **-0.13** | 0.03 | <.001 | **-0.16** | 0.06 | .008 |
| Emerging adulthood | **-0.99** | 0.13 | <.001 | **-0.96** | 0.22 | <.001 | | **-1.21** | 0.09 | <.001 | **-1.26** | 0.17 | <.001 |
| Established adulthood | **-0.89** | 0.14 | <.001 | **-0.79** | 0.24 | .001 | | **-1.54** | 0.10 | <.001 | **-1.64** | 0.18 | <.001 |
| Later-born cohort | **0.43** | 0.10 | <.001 | 0.45 | 0.25 | .077 | | -0.04 | 0.07 | .620 | -0.17 | 0.19 | .378 |
| Time x emerging adulthood | 0.00 | 0.06 | .994 | 0.05 | 0.10 | .621 | | 0.11 | 0.04 | .011 | 0.10 | 0.08 | .173 |
| Time x established adulthood | 0.15 | 0.07 | .028 | 0.07 | 0.10 | .489 | | **0.22** | 0.05 | <.001 | **0.25** | 0.08 | <.001 |
| Time x later-born cohort | 0.02 | 0.05 | .710 | 0.07 | 0.08 | .417 | | **-0.14** | 0.03 | <.001 | -0.08 | 0.06 | .214 |
| Emerging adulthood x later-born cohort | -0.17 | 0.19 | .370 | -0.17 | 0.31 | .579 | | 0.33 | 0.13 | .015 | 0.48 | 0.23 | .038 |
| Established adulthood x later-born cohort | **-0.80** | 0.20 | <.001 | **-0.88** | 0.32 | .006 | | **0.31** | 0.14 | .029 | 0.34 | 0.23 | .145 |
| Single duration |  |  |  | 0.00 | 0.00 | .168 | |  |  |  | 0.00 | 0.00 | .673 |
| Gender |  |  |  | **0.57** | 0.13 | <.001 | |  |  |  | -0.08 | 0.09 | .409 |

*Note*. For singlehood satisfaction, Level 1 sample sizes ranged from *N* = 2,984 (with control variables) to *N* = 8,667 (without control variables). For life satisfaction, Level 1 sample sizes ranged from *N* = 3,035 to *N* = 8,801 (without control variables). Significant parameters are displayed in bold (*p* ≤ .01).

| **Table S14** |  | | | | | |  | | | | | | | |
| --- | --- | --- | --- | --- | --- | --- | --- | --- | --- | --- | --- | --- | --- | --- |
| *Model 3: Gender Effects on Singlehood Satisfaction and Life Satisfaction* | | | | | | | | | | | | | | |
|  | Singlehood satisfaction | | | | | | Life satisfaction | | | | | | | |
|  | Without control variables | | | With control variables | | | | Without control variables | | | With control variables | | | |
|  | *b* | *SE* | *p* | *b* | *SE* | *p* | | *b* | *SE* | *p* | *b* | *SE* | *p* |  |
| Intercept | **6.89** | 0.08 | <.001 | **6.59** | 0.18 | <.001 | | **8.09** | 0.06 | <.001 | **8.20** | 0.14 | <.001 |  |
| Time | **-0.16** | 0.04 | <.001 | -0.11 | 0.09 | .207 | | **-0.13** | 0.03 | <.001 | **-0.17** | 0.06 | .009 |  |
| Emerging adulthood | **-1.06** | 0.14 | <.001 | **-1.09** | 0.24 | <.001 | | **-1.28** | 0.10 | <.001 | **-1.36** | 0.19 | <.001 |  |
| Established adulthood | **-1.14** | 0.17 | <.001 | **-1.12** | 0.28 | <.001 | | **-1.68** | 0.12 | <.001 | **-1.79** | 0.21 | <.001 |  |
| Later-born cohort | 0.22 | 0.12 | .055 | 0.36 | 0.27 | .181 | | 0.02 | 0.08 | .768 | -0.15 | 0.20 | .468 |  |
| Gender | 0.18 | 0.12 | .125 | 0.11 | 0.26 | .678 | | -0.18 | 0.08 | .027 | -0.32 | 0.20 | .109 |  |
| Time x emerging adulthood | 0.00 | 0.06 | .999 | 0.05 | 0.10 | .589 | | 0.11 | 0.04 | .011 | 0.10 | 0.08 | .181 |  |
| Time x established adulthood | 0.15 | 0.07 | .025 | 0.08 | 0.10 | .426 | | **0.23** | 0.05 | <.001 | **0.25** | 0.08 | .001 |  |
| Time x later-born cohort | 0.02 | 0.05 | .672 | 0.07 | 0.08 | .399 | | **-0.14** | 0.03 | <.001 | -0.08 | 0.06 | .209 |  |
| Time x gender | -0.04 | 0.05 | .392 | -0.06 | 0.08 | .459 | | -0.01 | 0.03 | .756 | 0.02 | 0.06 | .748 |  |
| Emerging adulthood x later-born cohort | -0.20 | 0.19 | .292 | -0.23 | 0.31 | .471 | | 0.32 | 0.13 | .016 | 0.46 | 0.23 | .049 |  |
| Established adulthood x later-born cohort | **-0.80** | 0.20 | <.001 | **-0.94** | 0.32 | .003 | | 0.31 | 0.14 | .030 | 0.33 | 0.24 | .165 |  |
| Emerging adulthood x gender | 0.19 | 0.19 | .314 | 0.37 | 0.31 | .231 | | 0.17 | 0.13 | .184 | 0.27 | 0.23 | .236 |  |
| Established adulthood x gender | 0.47 | 0.20 | .016 | 0.73 | 0.32 | .021 | | 0.32 | 0.14 | .023 | 0.34 | 0.23 | .142 |  |
| Later-born cohort x gender | **0.42** | 0.14 | .003 | 0.29 | 0.25 | .246 | | -0.12 | 0.10 | .257 | 0.00 | 0.18 | .992 |  |
| Single duration |  |  |  | 0.00 | 0.00 | .111 | |  |  |  | 0.00 | 0.00 | .586 |  |

*Note.* For singlehood satisfaction, Level 1 sample sizes ranged from *N* = 2,984 (with control variables) to *N* = 8,664 (without control variables). For life satisfaction, Level 1 sample sizes ranged from *N* = 3,035 to *N* = 8,798 (without control variables). Significant parameters are displayed in bold (*p* ≤ .01).

| **Table S15** |  | | | | | |  | | | | | | |
| --- | --- | --- | --- | --- | --- | --- | --- | --- | --- | --- | --- | --- | --- |
| *Model 4: Extraversion Effect on Singlehood Satisfaction and Life Satisfaction* | | | | | | | | | | | | | |
|  | Singlehood satisfaction | | | | | | Life satisfaction | | | | | | |
|  | Without control variables | | | With control variables | | | | Without control variables | | | With control variables | | |
|  | *b* | *SE* | *p* | *b* | *SE* | *p* | | *b* | *SE* | *p* | *b* | *SE* | *p* |
| Intercept | **6.15** | 0.21 | <.001 | **5.71** | 0.43 | <.001 | | **6.56** | 0.15 | <.001 | **6.61** | 0.33 | <.001 |
| Time | -0.24 | 0.10 | .014 | -0.10 | 0.20 | .595 | | 0.02 | 0.07 | .729 | -0.08 | 0.15 | .582 |
| Emerging adulthood | **-0.95** | 0.13 | <.001 | **-0.90** | 0.22 | <.001 | | **-1.15** | 0.09 | <.001 | **-1.14** | 0.16 | <.001 |
| Established adulthood | **-0.85** | 0.14 | <.001 | **-0.73** | 0.24 | .002 | | **-1.50** | 0.10 | <.001 | **-1.52** | 0.18 | <.001 |
| Later-born cohort | **1.01** | 0.28 | <.001 | 0.52 | 0.59 | .378 | | **0.63** | 0.20 | .001 | 0.10 | 0.42 | .816 |
| Extraversion | **0.23** | 0.06 | <.001 | 0.20 | 0.11 | .064 | | **0.43** | 0.04 | <.001 | **0.41** | 0.08 | <.001 |
| Time x emerging adulthood | 0.00 | 0.06 | .993 | 0.05 | 0.10 | .653 | | 0.11 | 0.04 | .014 | 0.10 | 0.08 | .203 |
| Time x established adulthood | 0.14 | 0.07 | .032 | 0.07 | 0.10 | .503 | | **0.22** | 0.05 | <.001 | **0.25** | 0.08 | .002 |
| Time x later-born cohort | 0.02 | 0.05 | .714 | 0.06 | 0.08 | .440 | | **-0.15** | 0.03 | <.001 | -0.08 | 0.06 | .194 |
| Time x extraversion | 0.02 | 0.03 | .567 | -0.01 | 0.05 | .896 | | -0.05 | 0.02 | .016 | -0.02 | 0.04 | .554 |
| Emerging adulthood x later-born cohort | -0.24 | 0.19 | .215 | -0.26 | 0.32 | .415 | | 0.21 | 0.13 | .112 | 0.32 | 0.23 | .158 |
| Established adulthood x later-born cohort | **-0.84** | 0.20 | <.001 | **-0.92** | 0.32 | .004 | | 0.25 | 0.14 | .070 | 0.23 | 0.23 | .315 |
| Later-born cohort x extraversion | -0.17 | 0.08 | .038 | 0.00 | 0.15 | .977 | | **-0.18** | 0.06 | .001 | -0.04 | 0.11 | .680 |
| Single duration |  |  |  | 0.00 | 0.00 | .108 | |  |  |  | 0.00 | 0.00 | .273 |
| Gender |  |  |  | **0.52** | 0.13 | <.001 | |  |  |  | -0.15 | 0.09 | .089 |

*Note*. For singlehood satisfaction, Level 1 sample sizes ranged from *N* = 2,963 (with control variables) to *N* = 8,640 (without control variables). For life satisfaction, Level 1 sample sizes ranged from *N* = 3,014 to *N* = 8,774 (without control variables). Significant parameters are displayed in bold (*p* ≤ .01).

**Table S16**

*Model 4: Neuroticism Effect on Singlehood Satisfaction and Life Satisfaction*

|  | Singlehood satisfaction | | | | | | Life satisfaction | | | | | | |
| --- | --- | --- | --- | --- | --- | --- | --- | --- | --- | --- | --- | --- | --- |
|  | Without control variables | | | With control variables | | | | Without control variables | | | With control variables | | |
|  | *b* | *SE* | *p* | *b* | *SE* | *p* | | *b* | *SE* | *p* | *b* | *SE* | *p* |
| Intercept | **8.41** | 0.17 | <.001 | **7.79** | 0.31 | <.001 | | **9.85** | 0.12 | <.001 | **9.97** | 0.23 | <.001 |
| Time | -0.15 | 0.08 | .072 | -0.02 | 0.15 | .912 | | **-0.24** | 0.06 | <.001 | -0.25 | 0.12 | .032 |
| Emerging adulthood | **-0.95** | 0.13 | <.001 | **-0.91** | 0.21 | <.001 | | **-1.16** | 0.09 | <.001 | **-1.20** | 0.16 | <.001 |
| Established adulthood | **-0.79** | 0.14 | <.001 | **-0.75** | 0.23 | .001 | | **-1.43** | 0.10 | <.001 | **-1.57** | 0.17 | <.001 |
| Later-born cohort | -0.13 | 0.25 | .615 | 0.39 | 0.47 | .412 | | -0.17 | 0.17 | .303 | -0.19 | 0.33 | .572 |
| Neuroticism | **-0.56** | 0.06 | <.001 | **-0.55** | 0.11 | <.001 | | **-0.70** | 0.04 | <.001 | **-0.75** | 0.08 | <.001 |
| Time x emerging adulthood | 0.00 | 0.06 | .983 | 0.05 | 0.10 | .591 | | 0.11 | 0.04 | .012 | 0.10 | 0.08 | .184 |
| Time x established adulthood | 0.14 | 0.07 | .033 | 0.08 | 0.10 | .451 | | **0.22** | 0.05 | <.001 | **0.25** | 0.08 | .002 |
| Time x later-born cohort | 0.02 | 0.05 | .707 | 0.08 | 0.08 | .363 | | **-0.15** | 0.04 | <.001 | -0.08 | 0.06 | .188 |
| Time x neuroticism | -0.01 | 0.03 | .750 | -0.04 | 0.05 | .390 | | 0.04 | 0.02 | .042 | 0.03 | 0.04 | .383 |
| Emerging adulthood x later-born cohort | -0.22 | 0.19 | .229 | -0.22 | 0.31 | .473 | | 0.28 | 0.12 | .027 | 0.43 | 0.21 | .042 |
| Established adulthood x later-born cohort | **-0.88** | 0.20 | <.001 | **-0.86** | 0.31 | .006 | | 0.22 | 0.13 | .090 | 0.34 | 0.22 | .117 |
| Later-born cohort x neuroticism | **0.24** | 0.08 | .005 | 0.05 | 0.15 | .739 | | 0.11 | 0.06 | .056 | 0.05 | 0.10 | .621 |
| Single duration |  |  |  | 0.00 | 0.00 | .123 | |  |  |  | 0.00 | 0.00 | .460 |
| Gender |  |  |  | **0.76** | 0.12 | <.001 | |  |  |  | 0.16 | 0.09 | .072 |

*Note*. For singlehood satisfaction, Level 1 sample sizes ranged from *N* = 2,966 (with control variables) to *N* = 8,643 (without control variables). For life satisfaction, Level 1 sample sizes ranged from *N* = 3,017 to *N* = 8,777 (without control variables). Significant parameters are displayed in bold (*p* ≤ .01).

| **Table S17** |  | | | | | |  | | | | | | | |
| --- | --- | --- | --- | --- | --- | --- | --- | --- | --- | --- | --- | --- | --- | --- |
| *Model 4: Big Five Personality Effects on Singlehood Satisfaction and Life Satisfaction* | | | | | | | | | | | | | | |
|  | Singlehood satisfaction | | | | | | Life satisfaction | | | | | | | |
|  | Without control variables | | | With control variables | | | | Without control variables | | | With control variables | | | |
|  | *b* | *SE* | *p* | *b* | *SE* | *p* | | *b* | *SE* | *p* | *b* | *SE* | *p* |  |
| Intercept | **8.00** | 0.46 | <.001 | **8.79** | 0.85 | <.001 | | **7.68** | 0.31 | <.001 | **7.76** | 0.61 | <.001 |  |
| Time | -0.46 | 0.22 | .035 | -0.23 | 0.40 | .570 | | -0.06 | 0.16 | .682 | -0.15 | 0.30 | .625 |  |
| Emerging adulthood | **-0.98** | 0.13 | <.001 | **-0.91** | 0.22 | <.001 | | **-1.22** | 0.09 | <.001 | **-1.29** | 0.16 | <.001 |  |
| Established adulthood | **-0.87** | 0.14 | <.001 | **-0.74** | 0.24 | .002 | | **-1.56** | 0.10 | <.001 | **-1.70** | 0.17 | <.001 |  |
| Later-born cohort | -0.13 | 0.63 | .843 | -0.29 | 1.19 | .809 | | -0.08 | 0.41 | .845 | -1.13 | 0.81 | .161 |  |
| Extraversion | 0.10 | 0.06 | .097 | 0.11 | 0.11 | .353 | | **0.23** | 0.04 | <.001 | 0.17 | 0.08 | .046 |  |
| Neuroticism | **-0.49** | 0.06 | <.001 | **-0.55** | 0.11 | <.001 | | **-0.57** | 0.04 | <.001 | **-0.63** | 0.08 | <.001 |  |
| Agreeableness | -0.03 | 0.07 | .666 | -0.24 | 0.12 | .036 | | 0.09 | 0.04 | .053 | 0.00 | 0.08 | .989 |  |
| Conscientiousness | **0.19** | 0.07 | .007 | 0.06 | 0.13 | .626 | | **0.31** | 0.05 | <.001 | **0.40** | 0.09 | <.001 |  |
| Openness | **-0.19** | 0.07 | .009 | -0.23 | 0.13 | .071 | | -0.08 | 0.05 | .104 | -0.01 | 0.09 | .886 |  |
| Time x emerging adulthood | -0.02 | 0.06 | .742 | 0.04 | 0.10 | .689 | | **0.12** | 0.04 | .009 | 0.13 | 0.08 | .100 |  |
| Time x established adulthood | 0.12 | 0.07 | .081 | 0.07 | 0.11 | .505 | | **0.23** | 0.05 | <.001 | **0.28** | 0.08 | .001 |  |
| Time x later-born cohort | 0.02 | 0.05 | .678 | 0.07 | 0.08 | .420 | | **-0.15** | 0.04 | <.001 | -0.09 | 0.06 | .172 |  |
| Time x extraversion | 0.00 | 0.03 | .958 | -0.05 | 0.05 | .318 | | -0.04 | 0.02 | .090 | 0.00 | 0.04 | .907 |  |
| Time x neuroticism | 0.00 | 0.03 | .878 | -0.07 | 0.05 | .208 | | 0.03 | 0.02 | .172 | 0.03 | 0.04 | .493 |  |
| Time x agreeableness | -0.02 | 0.03 | .557 | -0.02 | 0.06 | .672 | | 0.03 | 0.02 | .250 | 0.07 | 0.04 | .082 |  |
| Time x conscientiousness | 0.06 | 0.03 | .085 | -0.01 | 0.06 | .823 | | -0.03 | 0.02 | .223 | -0.08 | 0.05 | .082 |  |
| Time x openness | 0.04 | 0.03 | .258 | **0.16** | 0.06 | .009 | | 0.00 | 0.02 | .939 | 0.00 | 0.05 | .929 |  |
| Emerging adulthood x later-born cohort | -0.19 | 0.19 | .303 | -0.23 | 0.31 | .458 | | **0.32** | 0.12 | .010 | 0.48 | 0.21 | .023 |  |
| Established adulthood x later-born cohort | -0.83 | 0.20 | <.001 | -0.84 | 0.33 | .010 | | 0.26 | 0.13 | .043 | 0.30 | 0.22 | .179 |  |
| Later-born cohort x extraversion | -0.12 | 0.09 | .146 | -0.01 | 0.16 | .954 | | **-0.15** | 0.06 | .005 | -0.03 | 0.11 | .813 |  |
| Later-born cohort x neuroticism | 0.20 | 0.09 | .025 | 0.08 | 0.16 | .629 | | 0.07 | 0.06 | .254 | 0.06 | 0.11 | .602 |  |
| Later-born cohort x agreeableness | 0.04 | 0.09 | .647 | 0.07 | 0.17 | .666 | | 0.17 | 0.06 | .005 | **0.35** | 0.11 | .002 |  |
| Later-born cohort x conscientiousness | -0.06 | 0.10 | .581 | 0.00 | 0.19 | .997 | | -0.01 | 0.07 | .890 | -0.06 | 0.13 | .622 |  |
| Later-born cohort x openness | 0.16 | 0.10 | .111 | 0.11 | 0.18 | .561 | | 0.02 | 0.07 | .792 | 0.06 | 0.12 | .622 |  |
| Single duration |  |  |  | 0.00 | 0.00 | .093 | |  |  |  | 0.00 | 0.00 | .374 |  |
| Gender |  |  |  | **0.77** | 0.13 | <.001 | |  |  |  | 0.01 | 0.09 | .907 |  |

*Note*. For singlehood satisfaction, Level 1 sample sizes ranged from *N* = 2,963 (with control variables) to *N* = 8,637(without control variables). For life satisfaction, Level 1 sample sizes ranged from *N* = 3,014 to *N* = 8,771 (without control variables). Significant parameters are displayed in bold (*p* ≤ .01).
